# Comparative effects of different types of soy products on glycemic control and insulin sensitivity: a network meta-analysis of randomized controlled trials

**DOI:** 10.3389/fnut.2025.1681229

**Published:** 2026-01-20

**Authors:** Qiuping Luo, Baoguo Kang, Lifu Lei, Hongjia Yan, Mengting Chen, Ting Peng, Yuanlian Ouyang, Hailan Sun, Suocheng Hui

**Affiliations:** 1 Department of Clinical Nutrition, Liangjiang Hospital of Chongqing Medical University, Chongqing, China; 2 Department of Oncology, Liangjiang Hospital of Chongqing Medical University, Chongqing, China; 3 Department of Clinical Nutrition, The Second Affliated Hospital of Chongqing Medical University, Chongqing, China; 4 Department of Clinical Nutrition, Chongqing University Cancer Hospital, School of Medicine, Chongqing University, Chongqing, China; 5 Department of Clinical Nutrition, Women and Children's Hospital of Chongqing Medical University (Chongqing Health Center for Women and Children), Chongqing, China

**Keywords:** diabetes, glycemic control, insulin sensitivity, network meta-analysis, soy

## Abstract

**Background and aims:**

Accumulating evidence has demonstrated the benefits of soy and its extracts on glycemic control and insulin sensitivity. We hypothesized that different soy components might exhibit differentiated blood glucose and insulin sensitivity regulation effects. The goal of this network meta-analysis of randomized clinical trials (RCTs) was to estimate and rank the relative effects of soy and its extracts on glycemic control and insulin sensitivity.

**Methods:**

We performed a strategic literature search of PubMed, Embase, and the Cochrane Library for relevant RCTs. Random-effects network meta-analyses, ranking analyses based on surface under the cumulative ranking curves (SUCRAs), and sensitivity analyses based on potential sources of heterogeneity were performed. We registered the study protocol at PROSPERO (no. CRD42022345831).

**Results:**

Sixty-one RCTs enrolling 4,744 participants were included in the quantitative analysis. In random-effects network meta-analyses. Whole soy was ranked as the best diet (SUCRA: 91.0%) regarding fasting glucose-lowering effects, with isolated isoflavones was ranked as second (SUCRA: 79.1%). For fasting insulin regulation, the best soy component choices was whole soy (SUCRA: 95.4%) and isolated isoflavones (SUCRA: 74.7%). For HOMA-IR regulation, the best soy component choices was whole soy (SUCRA: 83.4%) and Soy protein + isoflavones (SUCRA: 83.3%). No soy products showed significant effect on regulating glycated HbA1c.

**Conclusions:**

These findings highlight that different soy components exhibit distinct effects on regulating blood glucose and insulin sensitivity. In this network meta-analysis, whole soy and isolated isoflavones were identified as potentially superior choices for improving blood glucose control and insulin sensitivity compared with other soy products.

**Systematic review registration:**

http://www.crd.york.ac.uk/ PROERO, identifier CRD42022345831.

## 1 Introduction

Type 2 diabetes (T2DM), manifested with hyperglycemia and insulin resistance, is now a prevalent disease of concern worldwide and represents the seventh leading cause of death. The global number of diabetes cases is estimated to increase to 629 million by 2045 (1). Mounting evidence has demonstrated that dietary modification is an important lifestyle approach for decreasing diabetes risk because of its favorable effects on improving hyperglycemia and insulin sensitivity (2–6). Due to the effects of a soy-based diet in reducing total cholesterol (TC), LDL cholesterol, triglycerides (TGs), body weight and postprandial glycemia, and several nutritional guidelines have also suggested increasing the dietary intake of soy products for the prevention and management of T2DM (7).

The favorable impact of soy products on T2DM is mostly due to its components such as soy protein, isoflavones (e.g., genistein, daidzein, and glycitein), polyunsaturated fatty acids, fiber, vitamins, minerals, and phytochemicals (8). Previous human studies have reported that soy products and extracts may have a positive impact on glucose control and insulin sensitivity (9–11). Compared with the control diet group, a 6-week soy protein intervention in women with gestational diabetes mellitus significantly reduced plasma glucose and serum insulin levels, with a concomitant improvement in the quantitative insulin sensitivity check index. Another randomized, double-blind, placebo-controlled trial confirmed that 100 mg/day soy isoflavones treatment for 8 weeks could significantly reduce blood glucose and insulin levels (12). However, the results are still inconsistent, in the meta-analysis by Tang et al. (13), a reduced risk of T2DM was associated with dietary intakes of soy protein, and isoflavones, but not with total legumes or whole soy. Furthermore, another cross-sectional study did not find any association between isoflavones and glycated hemoglobin (14). The above evidence suggests that soy and its different components have variable results, and it is inconclusive which type is superior to the others in terms of glycemic control and insulin sensitivity. Research to elucidate the different health effects of individual soy components is essential for formulating personalized diabetes management strategies. Thus, to address this gap in knowledge, we conducted a comprehensive systematic review and network meta-analysis of published RCTs to evaluate the comparative effects of consuming different soy products consumption on glucose control and insulin sensitivity.

## 2 Methods

The network meta-analysis was performed in accordance with the PRISMA (Preferred Reporting Items for Systematic Review and Meta-Analysis) guidelines (15). The research was registered at www.crd.york.ac.uk/ PROERO as number CRD42022345831.

### 2.1 Search strategy

We searched PubMed (http://www.ncbi.nlm.nih.gov/pubmed/), Embase (http://www.embase.com/search/advanced/), and the Cochrane Library (http://www.cochrane.org/) for clinical trials to March 2025. The retrieval strategy for the present study was as follows: (soy OR soya OR soybean OR soy protein OR isoflavone OR genistein OR daidzein) AND (glucose OR glycemic control OR glycaemic control OR glucose control OR insulin OR insulin sensitivity). To avoid missing relevant literature, we did not apply restrictions on article types during the search. The study searches were conducted separately by three investigators (QL, BK, and LL), and any disagreements were resolved through consultation with other co-authors (HS and SH).

### 2.2 Study selection

Studies were selected based on the following criteria: (1) Clinical trials comparing ≥2 of the following intervention components: whole soy, isolated isoflavones, soy protein, soy protein + isoflavones and control group (habitual diet or average local diet with no treatment foods or free of soy extracts supplementation); (2) Human participants in RCTs with parallel or crossover-design; (3) The duration of medium/long-term intervention was ≥2 weeks; (4) Reported end-point values for blood glucose, fasting insulin, homeostasis model assessment of insulin resistance (HOMA-IR), and glycosylated hemoglobin (HbA1c) in the control and intervention groups, along with standard error or standard deviation or 95% confidence intervals (CIs); (5) There were no special restrictions on the participants' health status; and (6) Articles published in English on PubMed, Embase or the Corhrance library.

### 2.3 Data extraction

We extracted the following study characteristics: (1) Research characteristics such as publication year, author information, study design, treatment duration, and sample size were extracted; (2) Demographic characteristics, such as age, percentage of female subjects, body mass index (BMI), and participants' health status; (3) Fasting blood glucose, plasma insulin, HOMA-IR and HbA1c at baseline and post-treatment; and (4) Dietary information/foods provided in both the intervention and control groups. The reported values is converted into unified measurement units to standardize the data. The three investigators (QL, BK, and LL) extracted data and relevant information from each trial independently, and any disagreements were settled through consultation with co-authors (HS and SH).

### 2.4 Risk of bias and quality of evidence

We used the Cochrane Bias Risk Tool to evaluate the bias risk of the included studies (16). The evaluation covered the following six items: selection bias, performance bias, detection bias, attrition bias, reporting bias, and other bias. If ≥1 item was assessed as high risk, the study was classified as high risk, and if all items were assessed as low risk, the study was classified as low risk. All other trials were classified as having a moderate or unclear risk of bias. The quality of evidence for all results was evaluated using the Grading of Recommendations Assessment, Development and Evaluation (GRADE) approach (17). To ensure the objectivity and accuracy, two researchers (QL and BK) independently reviewed enrolled studies and evaluated the risk of bias and the quality of evidence. Discrepancies were adjudicated by a third co-author (HS or SH).

### 2.5 Dealing with missing data

We attempted to contact the corresponding authors to obtain missing outcome data. When these data could not be obtained, the studies were excluded for failing to meet the inclusion criteria. According to the guidelines of the Cochrane Handbook using Review Manager software, SD values were calculated from SEs, 95% CIs, *P* values, or t statistics when they were not directly available.

### 2.6 Statistical analysis

We used Stata 14.1 software to perform network meta-analysis. The baseline and post-intervention values of outcomes used in the network meta-analysis are shown in Supplementary Table 1. The outcome measures in this paper were continuous variables, the mean difference (MD) and 95% CI were used as effect measures, and *I*^2^ was used to assess the magnitude of heterogeneity. We assessed the local inconsistency of included studies using the loop-specific approach and node-splitting method (18, 19). Simultaneously, we fitted the design-by-treatment interaction model to investigate the presence of inconsistency jointly from all possible sources in the network (20, 21). We used funnel plots to visually assess publication bias. To assess the robustness of the results, we performed a sensitivity analysis by fitting inconsistency models (22). We also conducted a sensitivity analysis to explore the impact of study design on the results. To rank and compare various interventions, the surface under the cumulative ranking (SUCRA) method was used (23). An intervention was included in the ranking only if at least three articles on the same soy extract were available. When SUCRA = 1, the intervention was considered completely effective; when SUCRA = 0, it was considered completely ineffective.

## 3 Results

### 3.1 Results of the literature search

**Figure 1:**
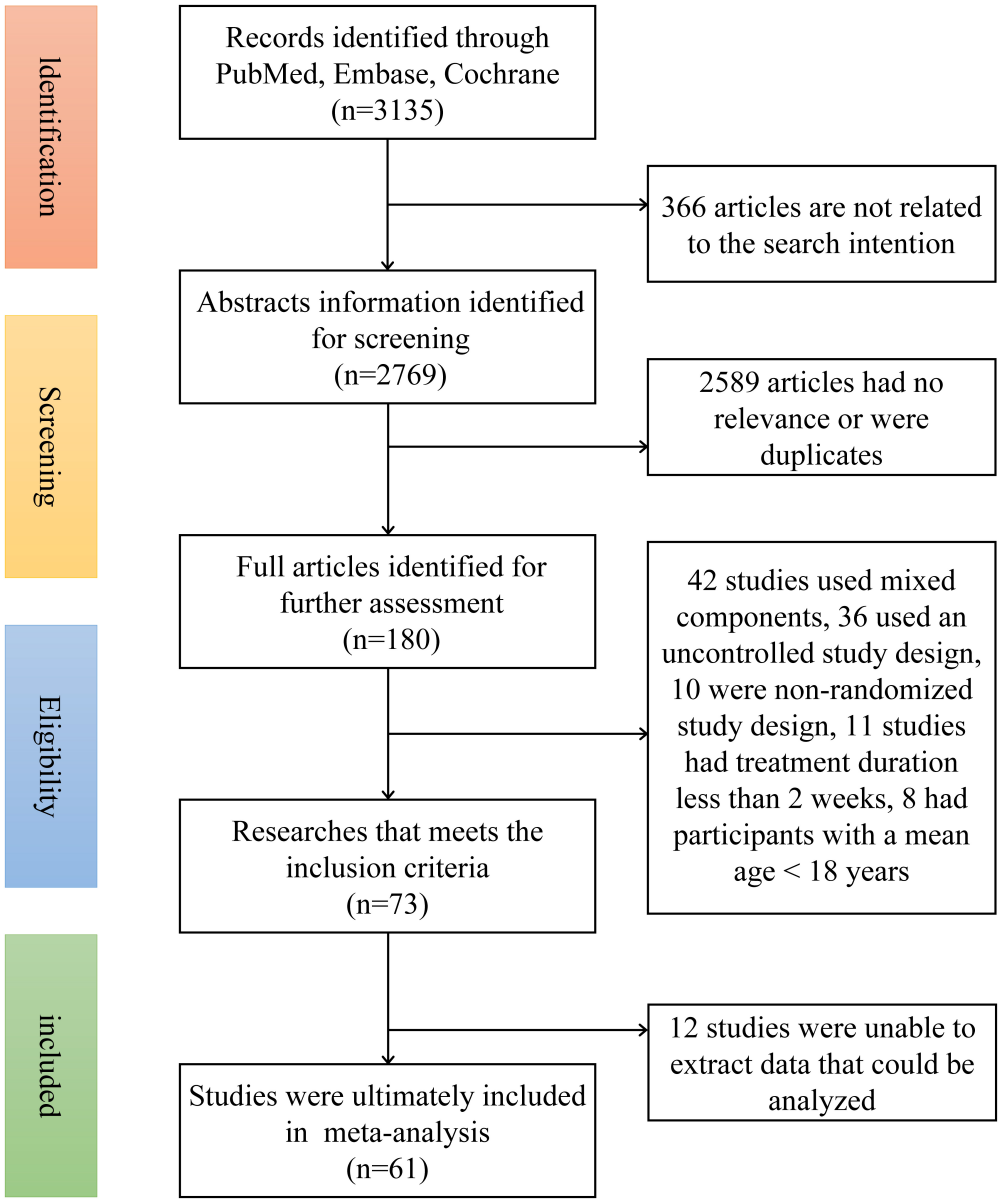
Flow diagram for study selection.

The flow chart for the search strategy and study selection is depicted in Figure 1. Initially, 3,135 articles were identified. Of these, 2,955 articles were excluded because they were irrelevant to the current meta-analysis or were duplicates. Therefore, 180 articles remained for further screening. Of these 180 articles, an additional 119 articles were excluded for the following reasons: 42 studies used mixed components, and 36 used an uncontrolled study design. Data from 12 studies were incomplete, 10 studies had a non-randomized study design, 11 studies had treatment duration less than 2 weeks, and eight studies had participants with a mean age less than 18 years. Overall, 61 articles were ultimately included in this meta-analysis.

### 3.2 Study characteristics

A total of 4,744 subjects were enrolled in this network meta-analysis. The included trials' sample sizes ranged from 11 to 389 participants. The mean age of the participants ranged from 24.2 to 69.5 years, and their BMI ranged from 23.3 to 36.1 kg/m^2^. The study included 61 trials, 17 of which studied healthy people with normal blood glucose levels and 45 of which studied people with metabolic syndrome and abnormal blood glucose levels. Among the 61 studies, there were 50 parallel trials and 11 cross-trial studies. More detailed study characteristics are displayed in Supplementary Table 2.

**Figure 2:**
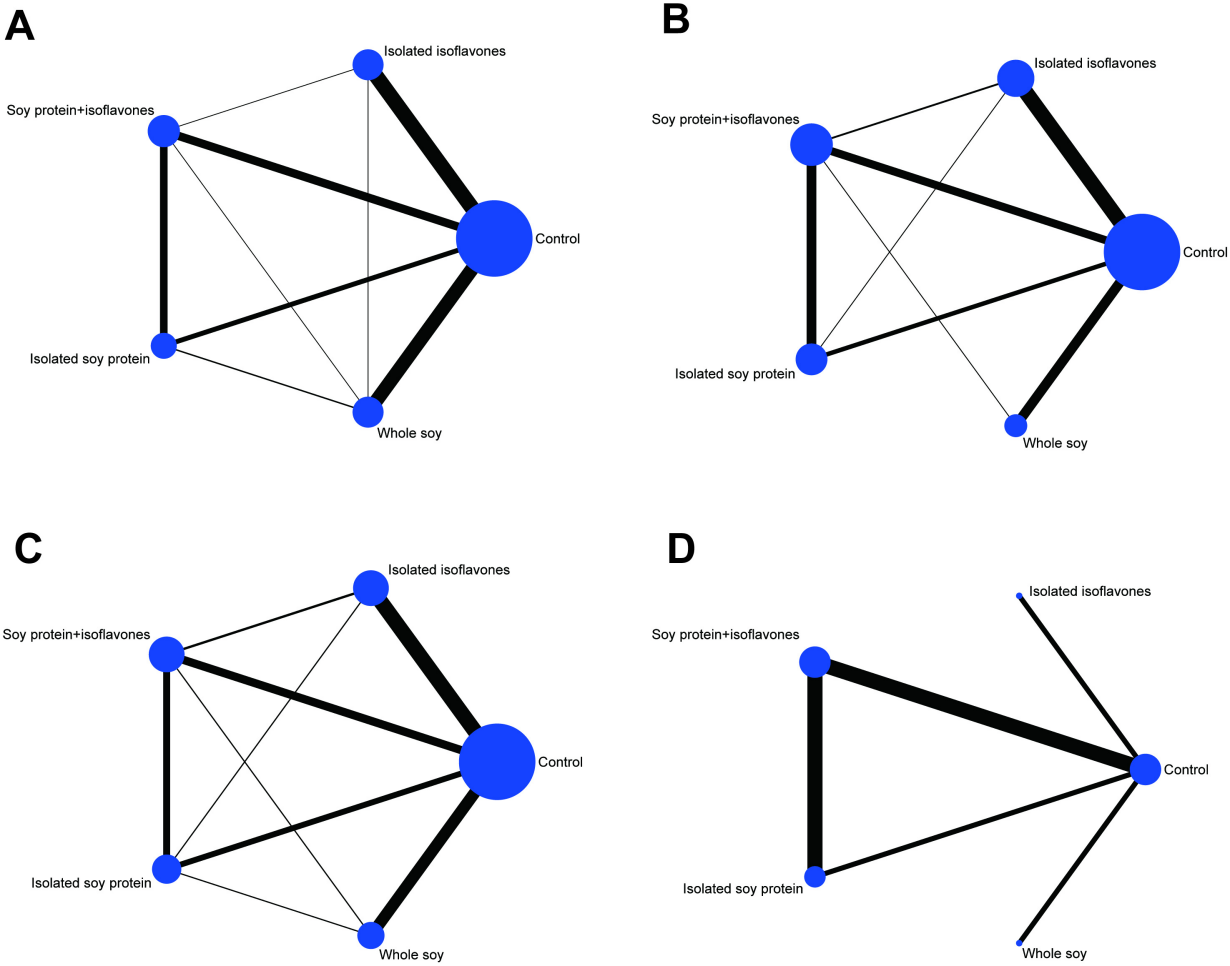
Network plots of eligible comparisons for different types of soy products for fasting glucose **(A)**, fasting insulin **(B)**, HOMA-IR **(C)**, and HbA1c **(D)**. Lines connect the interventions that have been studied in head-to-head comparisons in the eligible studies. The sizes of the nodes are proportional to the number of participants allocated to each diet group, and line thickness is proportional to the number of studies involved in each comparison.

The network relationship between the various interventions (i.e., different soy extracts) are depicted in Figure 2. The findings revealed that the majority of the eligible trials compared isolated isoflavones and whole soy with the control group, and there were several comparative studies among four different types of soy extracts. For effect indicator of HbA1c, no trial compared the intervention effect between whole soy with other soy extracts. Moreover, there was no comparison of intervention effects between soy protein and isolated isoflavones. Detailed results of the transitivity analyses are shown in Supplementary Figures 1–4. Contributions of direct evidence to the network analysis are reported in Supplementary Tables 3–6.

### 3.3 Risk of bias and quality of evidence

Overall, the risk of bias of included studies in the present meta-analysis was generally low or unclear (Supplementary Table 7, Supplementary Figures 5, 6). The majority of studies demonstrated a low risk of bias in the domains of incomplete outcome data and selective reporting. However, performance bias and detection bias were the primary concerns. Specifically, fourteen studies had a high risk of bias regarding the blinding of participants and personnel (performance bias), and four studies had a high risk of bias in the blinding of outcome assessment (detection bias). The risks in other domains, such as random sequence generation and allocation concealment, were predominantly low or unclear.

### 3.4 Network meta-analysis

#### 3.4.1 Fasting plasma glucose

**Figure 3:**
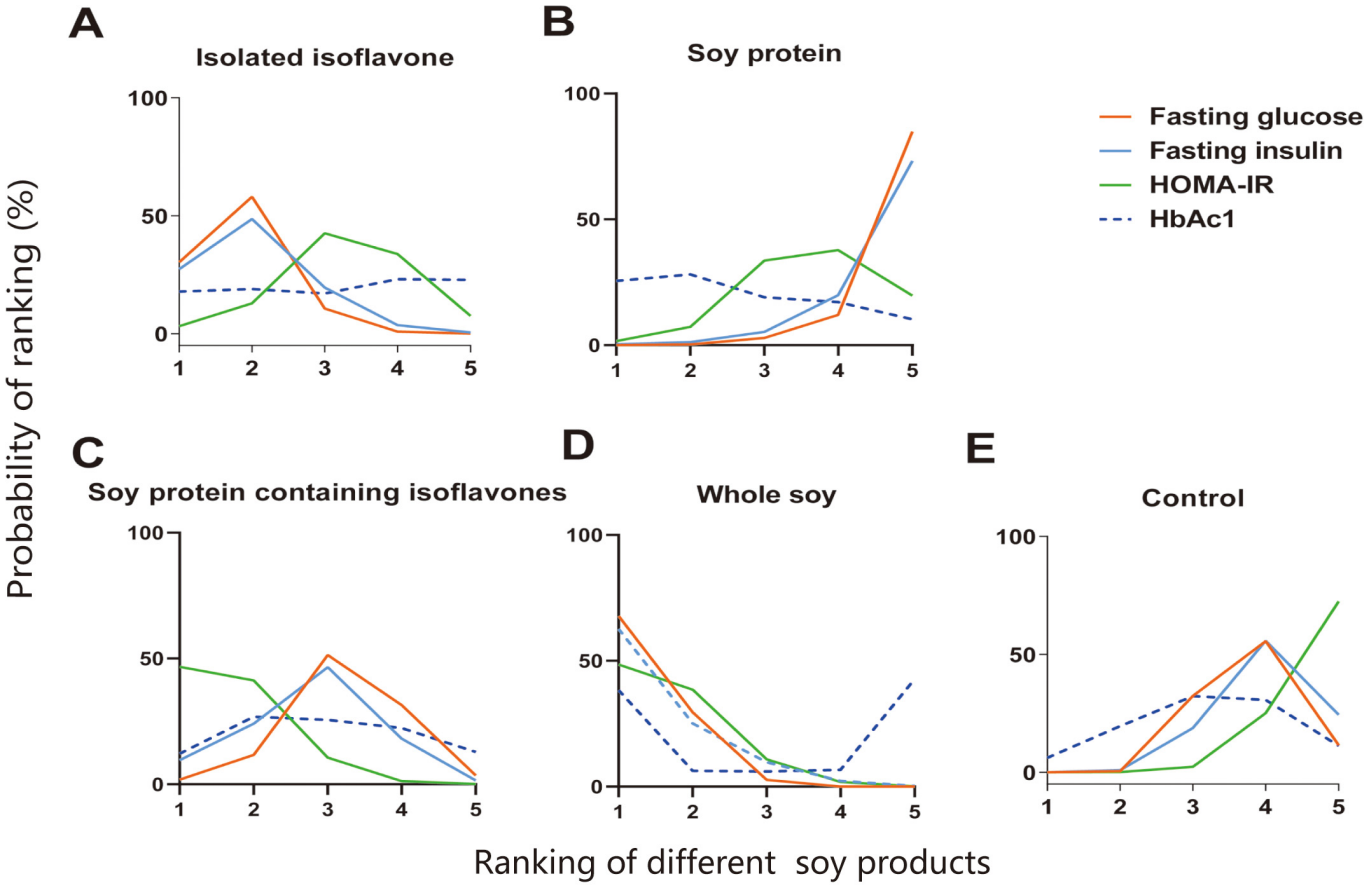
Ranking results of different types of soy products on glycemic control and insulin sensitivity. **(A)** Isolated isoflavone; **(B)** Soy protein; **(C)** Soy protein containing isoflavones; **(D)** Whole soy; **(E)** Control. The *x*-axis represents the ranking of interventions in numerical order, with the first representing the best. The *y*-axis represents the probability of each ranking.

Whole soy was ranked as the best diet (SUCRA: 91.0%) regarding blood glucose-lowering effects, followed by isoflavones (SUCRA: 79.1%; Figure 3, Supplementary Table 8). Compared with the control diet, significant blood glucose reductions were observed with the whole soy-enriched diet (−0.20 mmol/L, 95% CI: −0.33, −0.08) and isoflavones (−0.16 mmol/L, 95% CI: −0.30, −0.02; Table 1). The other soy extracts, soy protein and soy + isoflavones did not show significant effects on blood glucose compared with the control diet (Table 1). Whole soy intervention significantly decreased blood glucose concentration compared to soy protein (−0.30 mmol/L, 95% CI: −0.50, −0.10). Similarly, isoflavones alone also led to a significant reduction compared to the combination of Soy protein (−0.27 mmol/L, 95% CI: −0.47, −0.04; Table 1).

**Table 1:** Comparative effects of different types of soy products on the control of blood glucose.^*^

**Comparisons**	**Control**	**Whole soy**	**Soy protein**	**Soy protein + isoflavones**	**Isoflavones**
Control	–	**−0.20 (−0.33**, **−0.08)**	0.10 (−0.07, 0.27)	−0.04 (−0.19, 0.12)	**−0.16 (−0.30**, **−0.02)**
Whole soy		–	**0.30 (0.10, 0.50)**	0.17 (−0.02, 0.36)	0.05 (−0.14, 0.23)
Soy protein			–	−0.13 (−0.29, 0.02)	**−0.26 (−0.47**, **−0.04)**
Soy protein + isoflavones				–	−0.12 (−0.33, 0.08)
Isoflavones					–

^*^Mean differences with 95% confidence intervals (column vs. row). Mean differences lower than 0 indicate that the treatments specified in the row is more efficacious than those in the column. Bold values indicate statistically significant results (*p* < 0.05).

#### 3.4.2 Fasting plasma insulin

For fasting plasma insulin (FPI) reduction, whole soy (SUCRA: 87.0%) showed the highest probability to be the best diet, followed by isoflavones (SUCRA: 75.0%) and soy + isoflavones (SUCRA: 55.0%; Figure 3, Supplementary Table 9). In pooled analysis, soy + isoflavones (−0.92 mU/L, 95% CI: −1.84, −0.01) and whole soy intervention (−1.25 mU/L, 95% CI: −2.35, −0.16) significantly lowered FPI level compared with the control diet (Table 2). In addition, we found that, when compared with soy protein, whole soy intervention could significantly reduce the concentration of FPI (−1.72 mU/L, 95% CI: −3.41, −0.02; Table 2).

**Table 2:** Comparative effects of different types of soy products on the fasting insulin.^*^

**Comparisons**	**Control**	**Whole soy**	**Soy protein**	**Soy protein + isoflavones**	**Isoflavonse**
Control	–	**−1.25 (−2.35**, **−0.16)**	0.46 (−0.86, 1.79)	−0.52 (−1.64, 0.61)	**−0.92 (−1.84**, **−0.01)**
Whole soy		–	**1.72 (0.02, 3.41)**	0.74 (−0.78, 2.26)	0.33 (−1.09, 1.76)
Soy protein			–	−0.98 (−2.05, 0.10)	−1.38 (−2.91, 0.14)
Soy protein + isoflavones				–	−0.41 (−1.76, 0.95)
Isoflavones					–

^*^Mean differences with 95% confidence intervals (column vs. row). Mean differences lower than 0 indicate that the treatments specified in the row is more efficacious than those in the column. Bold values indicate statistically significant results (*p* < 0.05).

#### 3.4.3 HOMA-IR

As shown in Figure 3 and Supplementary Table 10, the results of the network meta-analysis on HOMA-IR demonstrated that whole soy (SUCRA: 83.3%) was the highest ranked treatment strategy for the reduction of HOMA-IR, followed by the soy protein + isoflavones (SUCRA: 82.8%). Compared with the control diet, soy protein + isoflavones (−0.50, 95% CI: −0.85, −0.14) and the whole soy-enriched diet (−0.51, 95% CI: −0.82, −0.19) significantly reduced HOMA-IR (Supplementary Table 11).

#### 3.4.4 HbA1c

The evaluated effects of different soy products on HbA1c are presented in Figure 3 and Supplementary Table 12. Based on the estimation results of HbA1c, soy protein (SUCRA: 60.4%) seemed to be more effective than control, followed by soy + isoflavones intervention (SUCRA: 51.8%) for HbA1c reduction. However, in pooled analysis, there was no significant effect of any soy products for HbA1c reduction (Supplementary Table 13).

#### 3.4.5 Assessment of inconsistency

The analyses of design-by-treatment model did not detect any significant global inconsistency for fasting glucose (*P* = 0.8425), fasting insulin (*P* = 0.3881), HOMA-IR (*P* = 0.9813), and HbA1c (*P* = 0.1198). Also, the node-splitting and loop-specific approach did not show significant inconsistencies. Detailed results of the assessment for inconsistency are shown in Supplementary Tables 14–17 and Supplementary Figures 7–10.

### 3.5 Quality of evidence

Detailed results of the quality of evidence are presented in Supplementary Tables 18–21. According to the GRADE framework, the quality of evidence for fasting glucose, fasting insulin, HOMA-IR, and HbA1c was rated as low or very low (see Supplementary Tables 18–21). The low and very low quality of evidence ratings was mainly due to the inconsistency, imprecision, risk of bias, and indirectness.

### 3.6 Sensitivity analysis and publication bias

As presented in Supplementary Tables 22–36, sensitivity analyses were conducted. Concerning the reduction of fasting plasma glucose (FPG), the results of subgroup analyses by excluding crossover trials, including only studies with normal glucose baseline, including only studies with glucose baseline, including only studies with dyslipidemic mean baseline, and including only trials with overweight or obese subjects were generally similar to the overall findings of the network analyses (whole soy or isoflavones was the best rank). For different subgroups, the best food to enhance insulin sensitivity might be whole soy, soy protein + isoflavones, isoflavones or soy protein. Concerning the regulation effect of HOMA-IR, both whole soy or soy protein + isoflavones shown significant effects on the reduction on HOMA-IR and were ranked as best intervention. Of note, when only including the studies with abnormal glucose baseline, the whole soy diet was consistently ranked as the best diet regarding FBG, plasma insulin, and HOMA-IR reduction. As shown in Supplementary Figures 11–14, the comparison adjusted funnel plots for fasting glucose, fasting insulin, HOMA-IR, and HbAc1 appeared slightly asymmetric for the trials included in this study.

## 4 Discussion

In this study, we hypothesized that different soy components might exhibit distinct effects on regulating blood glucose and insulin sensitivity. Our results indicated that whole soy was ranked as the best diet regarding fasting glucose-lowering effects, with isolated isoflavones ranked second. For fasting insulin regulation, the best soy component choices were whole soy and isolated isoflavones. For HOMA-IR regulation, the best soy component choice was whole soy and soy protein + isoflavones. Moreover, sensitivity analyses showed that whole soy was consistently the highest ranked treatment strategy for the reduction of fasting glucose, fasting insulin, and HOMA-IR in participants with abnormal glucose and lipids levels at baseline. Our study demonstrated that whole soy and isolated isoflavones were more effective interventions than other strategies for improving glucose control and insulin sensitivity.

To our knowledge, this is the first network meta-analysis comparing soy products and their components for glycemic control and insulin sensitivity. The role of soy in improving fasting glucose, insulin, and HOMA-IR has been explored in several traditional meta-analyses with inconsistent results (24–27). These inconsistent results may be due to the fact that some studies included monomer components of soy for combined analysis. Our results are consistent with two previous meta-analyses showing that whole soybeans reduced fasting blood glucose, and we found that whole soy also improves insulin sensitivity (25, 27). Moreover, previous meta-analyses have demonstrated that soy isoflavones are superior to controls for glycemic control and improved insulin sensitivity, which is consistent with the results of this study (28, 29). Soy isoflavones significantly improved blood glucose, insulin and HOMA-IR in pre- and post-menopausal women, as well as fasting blood glucose in patients with diabetic nephropathy (28–33). However, the beneficial effects of soy protein remain controversial. A meta-analysis of 11 studies suggested that soy protein could lower fasting glucose, insulin, and HOMA-IR in patients with type 2 diabetes and metabolic syndrome (11, 34), but other studies did not find this difference. As mentioned above, the current findings are inconsistent and lack evidence comparing the effects of whole soy and its components on glycemic control and insulin sensitivity. In our present study, we pooled direct and indirect evidence in a network of included studies to obtain estimates of relative effects between different interventions, which is an extension of traditional meta-analysis. Our results found that a diet rich in whole soy had the most significant effect on lowering fasting blood glucose, insulin, and HOMA-IR compared to a control diet. These results were consistent with previous findings that whole soy improved blood glucose and insulin sensitivity more than isoflavones or soy protein (9, 25, 35). The favorable differences may be due to the effects or interactions of other components in soy, apart from isoflavones and soy proteins, such as fiber, saponins, polysaccharides, phytosterols, and unsaturated fatty acids (36). For instance, dietary fiber and polysaccharides may modulate gut microbiota to increase short-chain fatty acid production, thereby improving insulin sensitivity (37), while saponins may inhibit α-glucosidase activity and exhibit anti-inflammatory properties (38). Furthermore, different processing procedures during the preparation of soy products (e.g., fermentation, boiling, or extrusion) can affect the integrity of protein subunits or alter soy isoflavones or some other components of soy protein, which may also have an impact on the biological function of soy (36, 39). Therefore, future research should prioritize elucidating how specific food processing methods influence the bioavailability and efficacy of soy's bioactive compounds. A deeper understanding of the complex interactions between preparation techniques, component composition, and physiological effects represents a critical next step for developing precise, evidence-based dietary recommendations.

In terms of reducing fasting glucose levels, whole soy was ranked as the best diet. Furthermore, whole soy consistently secured the top ranking as the most effective treatment strategy for the regulation of fasting glucose, fasting insulin, and HOMA-IR in participants with abnormal glucose levels at baseline. Therefore, our data suggest that populations, especially people with diabetes, could achieve better blood glucose and insulin sensitivity improvements using a diet rich in whole soy than with individual soy components. Several potential mechanisms may be involved in the beneficial effects of soy on glycemic control and insulin sensitivity. First, soy isoflavones activate nuclear receptors, including peroxisome-proliferator activated receptors (PPAR) α, PPARγ, and PPARδ, to regulate lipid and glucose metabolism (40). Moreover, isoflavones enhance the activity of hepatic antioxidant enzymes, which in turn increases insulin sensitivity and improves glucose uptake by the muscles, as well as favorably affecting the metabolic and secretory activities of adipose tissue (41). Second, soy protein is beneficial for improving insulin sensitivity and glucose utilization due to its abundance of specific amino acids such as glycine and arginine. This specific amino acid pattern also increases the sensitivity of pancreatic β cells and stimulates insulin secretion (23, 42). Third, the hypoglycemic effect may also be attributed to other components of the whole soy (e.g., fiber, saponins, polysaccharides, phytosterols, etc.) or their interactions with soy protein or isoflavones (25, 43). For example, soyasaponins have inhibitory effects on alpha-glucosidase, which can reduce fasting blood glucose and improve insulin resistance (38). Additionally, individual responses, potentially influenced by genetic factors such as the ability of certain gut microbiota to produce the isoflavone-derived metabolite equol from daidzein, may also play a role (44). However, human evidence on the hypoglycemic effects of soy components other than isoflavones and protein are limited, so more studies are needed in the future to investigate the effects and mechanisms of these components.

In this study, the quality of the evidence was mostly low or very low. The main reason for the downgrading of quality was the inconsistency and imprecision. This primarily limits the confidence in the precise effect estimates and underscores the need for cautious interpretation. Some indicators lack or have few studies with direct comparisons of two interventions, and indirect comparisons may increase the imprecision of the analyzed results. Moreover, there was potential heterogeneity between included studies. Interestingly, we found that the whole soy diet was always ranked as the best diet regarding FBG, plasma insulin, and HOMA-IR reduction in the studies of participants with abnormal glucose levels at baseline. Furthermore, some other factors, such as dietary habits, medication treatment use and physical activity, may have contributed to heterogeneity, but were not be statistically analyzed because the data were not available.

Some limitations of this study should be considered. First, the generalizability of our findings may be limited. The evidence base is characterized by a predominance of studies conducted in East Asia, North America, and Europe, with underrepresentation of other regions, and a majority of female participants, leading to a lack of data on male populations. Variations in habitual soy intake, genetic background, and sex-specific metabolic responses between these groups could influence the outcomes. Thus, future studies from diverse regions and including more men are required. Second, the differences in treatment protocols (including a wide range of intervention doses and duration) of the included trials in this study may contribute to heterogeneity; however, the results of our heterogeneity analyses showed that low and moderate heterogeneity was acceptable. It should be noted that this variation also precluded a reliable dose-response meta-analysis, representing a significant gap in the current literature. Third, most of the studies included in our study were placebo-controlled trials, and the number of trials directly comparing different treatments remains limited. Future studies with direct comparison of treatments are needed to further confirm our results. Furthermore, the certainty of the evidence for almost all network estimates was rated as low to very low because of the serious risk of bias, imprecision, and inconsistency (45).

Despite these limitations, our findings hold practical significance. Our results support recommending whole soy foods for glycemic management. This recommendation aligns with international guidelines, such as those from the American Diabetes Association, which advocate for plant-based eating patterns (46, 47). To translate this into practice, incorporating 1–2 daily servings of traditional whole soy foods (e.g., 100 g of tofu, 240 ml of unsweetened soy milk, or 80 g of edamame) could be a feasible and beneficial strategy for individuals with or at risk for type 2 diabetes, providing approximately 50–100 mg of isoflavones as used in many efficacious studies (48, 49). In summary, the current network meta-analysis provides comprehensive evidence about the comparative effects of different soy components on glycemic control and insulin sensitivity. Whole soy and isolated soy isoflavones showed significant reductions in fasting blood glucose and insulin. Moreover, whole soy, soy protein + isoflavones, and isolated isoflavones were the most effective interventions for lowering fasting glucose, insulin, and HOMA-IR, respectively. Sensitivity analyses of baseline glycemic abnormalities indicate that whole soy is the best intervention for improving blood glucose and insulin sensitivity in patients with dysglycemia. Future research should prioritize high-quality RCTs with direct comparisons, diverse populations, and dose-ranging designs to establish definitive conclusions and explore the synergistic mechanisms of whole soy.

## Data Availability

The original contributions presented in the study are included in the article/Supplementary material, further inquiries can be directed to the corresponding authors.
