# Supplementary material for: Comparative effects of different types of soy products on glycemic control and insulin sensitivity: a network meta-analysis of randomized controlled trials
Source: Front Nutr. 2026 Jan 20;12:1681229. doi: 10.3389/fnut.2025.1681229 (PMC12864099; doi:10.3389/fnut.2025.1681229)
Supplement: Supplementary file 1 [file Data_Sheet_1.PDF]

Comparative effects of different types of soy products on glycemic control and insulin sensitivity: a network meta-analysis of randomized controlled trials

Qiuping Luo[1#], Baoguo Kang[3#], Kai Wang[4#], Lifu Lei[5], Hongjia Yan[1], Mengting Chen[6], Ting Peng[1], Yuanlian Ouyang[1], Hailan Sun[2*], Suocheng Hui[1*]

[1]Department of Clinical Nutrition, Liangjiang Hospital of Chongqing Medical University, Chongqing, China

[2]Department of Clinical Nutrition, Women and Children's Hospital of Chongqing Medical University (Chongqing Health Center for Women and Children), Chongqing, China

[3]Department of Oncology, Liangjiang Hospital of Chongqing Medical University, Chongqing, China

[4]Department of Research, The PLA Rocket Force Characteristic Medical Center, Beijing, China

[5]Department of Clinical Nutrition, The Second Affliated Hospital of Chongqing Medical University, Chongqing, China

[6]Department of Clinical Nutrition, Chongqing University Cancer Hospital, School of Medicine, Chongqing University, Chongqing, China

#Qiuping Luo, Baoguo Kang and Kai Wang contributed equally to this work.

* Corresponding authors:

Hailan Sun, E-mail: sunhailan@hospital.cqmu.edu.cn

Suocheng Hui, E-mail: Suocheng.hui@hotmail.com

Supplemental Methods

Search strategy

Pubmed

(((((((soy) OR (soya)) OR (soybean)) OR (soy protein)) OR (isoflavones)) OR (genistein)) OR (daidzein)) AND ((((((glucose) OR (glycemic control)) OR (glycaemic control)) OR (glucose control)) OR (insulin)) OR (insulin sensitivity)) AND (Randomized Controlled Trial [ptyp] OR Clinical Trial [ptyp])

Embase

(soy OR soya OR soybean OR soy protein OR isoflavones OR genistein OR daidzein) AND (glucose OR glycemic control OR glycaemic control OR glucose control OR insulin OR insulin sensitivity) AND [controlled clinical trial]/lim AND [embase]/lim

Cochrane Library

(soy OR soya OR soybean OR soy protein OR isoflavones OR genistein OR daidzein) AND (glucose OR glycemic control OR glycaemic control OR glucose control OR insulin OR insulin sensitivity) in All Text

SUPPLEMENTAL TABLE 1 Baseline and Outcome data used in the network meta-analysis.[1]

**First author, publication year, country**	**Group**	**N**	**Fasting blood glucose**		**Fasting blood insulin**		**HOMA-IR**		**HbA1c**	
Azadbakht, 2006, Iran(1)	Soy protein	42	Baseline	6.61±0.22	Baseline	Unavailable	Baseline	4.20±0.26	Baseline	Unavailable
			Outcome	6.17±0.32	Outcome	Unavailable	Outcome	3.60±0.19	Outcome	Unavailable
	Whole soy	42	Baseline	6.56±0.18	Baseline	Unavailable	Baseline	4.16±0.39	Baseline	Unavailable
			Outcome	5.72±0.18	Outcome	Unavailable	Outcome	3.30±0.19	Outcome	Unavailable
	Control	42	Baseline	6.67±0.22	Baseline	Unavailable	Baseline	4.19±0.19	Baseline	Unavailable
			Outcome	6.22±0.36	Outcome	Unavailable	Outcome	3.90±0.26	Outcome	Unavailable
Atteritano, 2007, Italy (2)	Isoflavone	198	Baseline	5.25±0.68	Baseline	7.24±2.39	Baseline	1.69±0.42	Baseline	Unavailable
			Outcome	5.00±0.47	Outcome	6.66±2.25	Outcome	1.48±0.42	Outcome	Unavailable
	Control	191	Baseline	5.29±0.56	Baseline	7.24±2.49	Baseline	1.70±0.55	Baseline	Unavailable
			Outcome	5.40±0.39	Outcome	7.95±2.63	Outcome	1.91±0.55	Outcome	Unavailable
Aubertin-Leheudre, 2008, Canada (3)	Isoflavone	21	Baseline	5.01±0.49	Baseline	7.63±3.35	Baseline	1.00±0.45	Baseline	Unavailable
			Outcome	4.91±0.48	Outcome	6.37±2.78	Outcome	0.98±0.78	Outcome	Unavailable
	Control	18	Baseline	4.96±0.59	Baseline	10.22±4.61	Baseline	1.37±0.61	Baseline	Unavailable
			Outcome	4.97±0.41	Outcome	7.24±3.82	Outcome	1.11±0.76	Outcome	Unavailable
Acharjee, 2015, USA (4)	Soy protein + isoflavone	11	Baseline	5.56±0.43	Baseline	Unavailable	Baseline	Unavailable	Baseline	Unavailable
			Outcome	5.44±0.47	Outcome	Unavailable	Outcome	Unavailable	Outcome	Unavailable
	Control	11	Baseline	5.56±0.43	Baseline	Unavailable	Baseline	Unavailable	Baseline	Unavailable
			Outcome	5.56±0.43	Outcome	Unavailable	Outcome	Unavailable	Outcome	Unavailable
Acharjee, 2015, USA (4)	Soy protein + isoflavone	49	Baseline	5.36±0.54	Baseline	Unavailable	Baseline	Unavailable	Baseline	Unavailable
			Outcome	5.43±0.59	Outcome	Unavailable	Outcome	Unavailable	Outcome	Unavailable
	Control	49	Baseline	5.36±0.54	Baseline	Unavailable	Baseline	Unavailable	Baseline	Unavailable
			Outcome	5.36±0.54	Outcome	Unavailable	Outcome	Unavailable	Outcome	Unavailable
Amanat,2018, Iran (5)	Isoflavone	41	Baseline	5.80±0.67	Baseline	16.18±3.23	Baseline	4.17±1.01	Baseline	Unavailable
			Outcome	5.78±0.69	Outcome	14.77±1.78	Outcome	3.78±0.73	Outcome	Unavailable
	Control	37	Baseline	5.82±1.61	Baseline	16.15±2.12	Baseline	4.25±1.74	Baseline	Unavailable
			Outcome	6.19±3.41	Outcome	15.99±1.73	Outcome	4.27±2.47	Outcome	Unavailable
Barsalani, 2013, Canada (6)	Isoflavone	26	Baseline	4.4±0.52	Baseline	6.19±5.06	Baseline	1.23±1.03	Baseline	Unavailable
			Outcome	4.6±0.78	Outcome	6.48±4.87	Outcome	1.40±1.30	Outcome	Unavailable
	Control	26	Baseline	4.5±0.54	Baseline	5.76±4.47	Baseline	1.21±1.07	Baseline	Unavailable
			Outcome	4.8±0.67	Outcome	5.18±4.28	Outcome	1.15±1.07	Outcome	Unavailable

SUPPLEMENTAL TABLE 1 Continued

First author, publication year, country	Group	N	Fasting blood glucose		Fasting blood insulin		HOMA-IR		HbA1c	
Beavers, 2015, USA (7)	Soy protein + isoflavone	12	Baseline	5.33±0.67	Baseline	14.20±9.80	Baseline	Unavailable	Baseline	Unavailable
			Outcome	5.26±0.44	Outcome	10.70±4.16	Outcome	Unavailable	Outcome	Unavailable
	Control	12	Baseline	5.44±0.39	Baseline	14.90±10.10	Baseline	Unavailable	Baseline	Unavailable
			Outcome	5.17±0.44	Outcome	9.50±4.16	Outcome	Unavailable	Outcome	Unavailable
Braxas,2019, Iran (8)	Isoflavone	28	Baseline	11.04±4.25	Baseline	Unavailable	Baseline	Unavailable	Baseline	Unavailable
			Outcome	9.12±3.87	Outcome	Unavailable	Outcome	Unavailable	Outcome	Unavailable
	Control	26	Baseline	10.90±3.05	Baseline	Unavailable	Baseline	Unavailable	Baseline	Unavailable
			Outcome	10.74±3.55	Outcome	Unavailable	Outcome	Unavailable	Outcome	Unavailable
Bakhtiari, 2019, Iran (9)	Whole soy	75	Baseline	5.82±0.97	Baseline	12.80±6.84	Baseline	3.40±2.17	Baseline	Unavailable
			Outcome	5.02±1.05	Outcome	10.80±6.84	Outcome	2.50±1.82	Outcome	Unavailable
	Soy protein + isoflavone	75	Baseline	5.79±1.12	Baseline	11.60±8.23	Baseline	3.00±2.34	Baseline	Unavailable
			Outcome	5.43±1.13	Outcome	10.80±7.36	Outcome	2.60±1.99	Outcome	Unavailable
	Control	75	Baseline	5.69±1.20	Baseline	13.40±7.79	Baseline	3.40±2.17	Baseline	Unavailable
			Outcome	5.53±1.36	Outcome	13.80±7.79	Outcome	3.40±2.08	Outcome	Unavailable
Colacurci, 2005, Italy(10)	Isoflavone	29	Baseline	4.70±0.20	Baseline	Unavailable	Baseline	Unavailable	Baseline	Unavailable
			Outcome	4.60±0.30	Outcome	Unavailable	Outcome	Unavailable	Outcome	Unavailable
	Control	28	Baseline	4.60±0.30	Baseline	Unavailable	Baseline	Unavailable	Baseline	Unavailable
			Outcome	4.70±0.40	Outcome	Unavailable	Outcome	Unavailable	Outcome	Unavailable
Crisafulli, 2005, Italy (11)	Isoflavone	30	Baseline	4.74±0.60	Baseline	7.00±3.01	Baseline	1.47±0.66	Baseline	Unavailable
			Outcome	4.3±0.54	Outcome	6.24±2.46	Outcome	1.18±0.44	Outcome	Unavailable
	Control	30	Baseline	5.00±0.66	Baseline	6.60±4.66	Baseline	1.45±1.10	Baseline	Unavailable
			Outcome	5.30±1.04	Outcome	8.23±3.89	Outcome	2.00±1.15	Outcome	Unavailable
Chan, 2008, China (12)	Isoflavone	50	Baseline	5.80±0.30	Baseline	6.70±4.20	Baseline	6.80±1.30	Baseline	Unavailable
			Outcome	-0.20±1.00	Outcome	0.50±3.20	Outcome	0.20±0.60	Outcome	Unavailable
	Control	52	Baseline	6.10±3.70	Baseline	6.20±3.70	Baseline	7.00±1.70	Baseline	Unavailable
			Outcome	-0.10±1.20	Outcome	1.50±3.70	Outcome	0.20±0.60	Outcome	Unavailable
Chang, 2008, Korea (13)	Soy protein	10	Baseline	9.43±4.04	Baseline	Unavailable	Baseline	Unavailable	Baseline	8.90±2.53
			Outcome	6.11±1.35	Outcome	Unavailable	Outcome	Unavailable	Outcome	7.10±0.95
	Control	10	Baseline	10.17±2.58	Baseline	Unavailable	Baseline	Unavailable	Baseline	8.10±0.95
			Outcome	9.61±1.72	Outcome	Unavailable	Outcome	Unavailable	Outcome	7.10±0.63

SUPPLEMENTAL TABLE 1 Continued

First author, publication year, country	Group	N	Fasting blood glucose		Fasting blood insulin		HOMA-IR		HbA1c	
Charles, 2009, USA (14)	Soy protein + isoflavone	32	Baseline	4.66±0.53	Baseline	5.62±3.11	Baseline	1.19±0.74	Baseline	Unavailable
			Outcome	4.70±0.40	Outcome	5.33±2.66	Outcome	1.13±0.62	Outcome	Unavailable
	Control	43	Baseline	4.78±0.76	Baseline	5.69±6.36	Baseline	1.33±2.23	Baseline	Unavailable
			Outcome	4.72±0.54	Outcome	5.23±3.41	Outcome	1.10±0.72	Outcome	Unavailable
Choquette, 2010, Canada (15)	Isoflavone	23	Baseline	4.87±0.46	Baseline	7.01±3.54	Baseline	1.56±0.89	Baseline	Unavailable
			Outcome	4.86±0.5	Outcome	6.49±3.2	Outcome	1.47±0.84	Outcome	Unavailable
	Control	22	Baseline	4.99±0.55	Baseline	9.56±5.04	Baseline	2.24±1.28	Baseline	Unavailable
			Outcome	4.99±0.39	Outcome	7.76±5.77	Outcome	1.74±1.39	Outcome	Unavailable
Chilibeck, 2013, Canada (16)	Isoflavone	76	Baseline	5.24±0.85	Baseline	Unavailable	Baseline	Unavailable	Baseline	Unavailable
			Outcome	-0.04±0.69	Outcome	Unavailable	Outcome	Unavailable	Outcome	Unavailable
	Control	73	Baseline	5.30±0.85	Baseline	Unavailable	Baseline	Unavailable	Baseline	Unavailable
			Outcome	0.00±0.70	Outcome	Unavailable	Outcome	Unavailable	Outcome	Unavailable
Duncan, 1999, USA (17)	Isoflavone	17	Baseline	Unavailable	Baseline	4.72±2.01	Baseline	Unavailable	Baseline	Unavailable
			Outcome	Unavailable	Outcome	3.90±0.90	Outcome	Unavailable	Outcome	Unavailable
	Control	9	Baseline	Unavailable	Baseline	4.72±2.01	Baseline	Unavailable	Baseline	Unavailable
			Outcome	Unavailable	Outcome	3.80±0.88	Outcome	Unavailable	Outcome	Unavailable
Duncan, 1999, USA (17)	Isoflavone	18	Baseline	Unavailable	Baseline	4.72±2.01	Baseline	Unavailable	Baseline	Unavailable
			Outcome	Unavailable	Outcome	3.71±0.88	Outcome	Unavailable	Outcome	Unavailable
	Control	9	Baseline	Unavailable	Baseline	4.72±2.01	Baseline	Unavailable	Baseline	Unavailable
			Outcome	Unavailable	Outcome	3.80±0.88	Outcome	Unavailable	Outcome	Unavailable
Deibert, 2011, Germany (18)	Soy protein	13	Baseline	5.50±1.01	Baseline	16.00±10.00	Baseline	4.00±2.70	Baseline	Unavailable
			Outcome	5.18±0.76	Outcome	11.70±6.00	Outcome	2.70±1.60	Outcome	Unavailable
	Control	13	Baseline	5.38±0.76	Baseline	9.70±11.4	Baseline	2.40±3.00	Baseline	Unavailable
			Outcome	5.17±0.46	Outcome	8.00±8.1	Outcome	1.90±2.20	Outcome	Unavailable
Garrido, 2006, Chile (19)	Isoflavone	15	Baseline	Unavailable	Baseline	9.33±0.88	Baseline	Unavailable	Baseline	Unavailable
			Outcome	Unavailable	Outcome	8.52±0.82	Outcome	Unavailable	Outcome	Unavailable
	Control	14	Baseline	Unavailable	Baseline	10.28±1.22	Baseline	Unavailable	Baseline	Unavailable
			Outcome	Unavailable	Outcome	9.88±0.98	Outcome	Unavailable	Outcome	Unavailable

SUPPLEMENTAL TABLE 1 Continued

First author, publication year, country	Group	N	Fasting blood glucose		Fasting blood insulin		HOMA-IR		HbA1c	
Gardner, 2007, USA (20)	Whole soy	28	Baseline	5.00±0.39	Baseline	Unavailable	Baseline	Unavailable	Baseline	Unavailable
			Outcome	5.17±0.50	Outcome	Unavailable	Outcome	Unavailable	Outcome	Unavailable
	Soy protein	28	Baseline	5.00±0.39	Baseline	Unavailable	Baseline	Unavailable	Baseline	Unavailable
			Outcome	5.06±0.61	Outcome	Unavailable	Outcome	Unavailable	Outcome	Unavailable
	Control	28	Baseline	5.00±0.39	Baseline	Unavailable	Baseline	Unavailable	Baseline	Unavailable
			Outcome	5.06±0.56	Outcome	Unavailable	Outcome	Unavailable	Outcome	Unavailable
Gonzalez, 2007, the UK (21)	Isoflavone	26	Baseline	6.90±1.30	Baseline	12.80±8.10	Baseline	4.03±3.30	Baseline	Unavailable
			Outcome	6.80±1.20	Outcome	14.10±9.20	Outcome	4.50±3.80	Outcome	Unavailable
	Control	26	Baseline	7.00±1.40	Baseline	14.10±10.00	Baseline	4.60±4.50	Baseline	Unavailable
			Outcome	6.90±1.30	Outcome	13.00±6.90	Outcome	4.50±2.50	Outcome	Unavailable
Gobert,2010, Canada (22)	Soy protein + isoflavone	29	Baseline	6.73±1.35	Baseline	Unavailable	Baseline	Unavailable	Baseline	5.92±0.69
			Outcome	7.01±1.40	Outcome	Unavailable	Outcome	Unavailable	Outcome	5.91±0.69
	Control	26	Baseline	6.92±1.40	Baseline	Unavailable	Baseline	Unavailable	Baseline	5.94±0.69
			Outcome	6.89±1.18	Outcome	Unavailable	Outcome	Unavailable	Outcome	5.97±0.63
Hermansen, 2001, DeUnavailableleark (23)	Soy protein + isoflavone	20	Baseline	6.90±2.30	Baseline	9.79±5.33	Baseline	Unavailable	Baseline	6.60±1.20
			Outcome	7.30±2.80	Outcome	10.66±6.48	Outcome	Unavailable	Outcome	6.60±1.20
	Control	20	Baseline	7.00±2.00	Baseline	10.37±4.32	Baseline	Unavailable	Baseline	6.70±1.30
			Outcome	7.70±2.90	Outcome	9.79±5.90	Outcome	Unavailable	Outcome	6.90±1.70
Han, 2002, Brazil (24)	Soy protein + isoflavone	40	Baseline	5.31±0.53	Baseline	Unavailable	Baseline	Unavailable	Baseline	Unavailable
			Outcome	5.41±0.49	Outcome	Unavailable	Outcome	Unavailable	Outcome	Unavailable
	Control	40	Baseline	5.38±0.60	Baseline	Unavailable	Baseline	Unavailable	Baseline	Unavailable
			Outcome	5.22±0.49	Outcome	Unavailable	Outcome	Unavailable	Outcome	Unavailable
Hermansen, 2005, DeUnavailableleark (25)	Soy protein + isoflavone	43	Baseline	5.50±0.60	Baseline	7.54±2.89	Baseline	1.29±0.56	Baseline	Unavailable
			Outcome	5.40±0.60	Outcome	6.47±2.75	Outcome	1.10±0.54	Outcome	Unavailable
	Control	46	Baseline	5.30±0.50	Baseline	7.53±3.47	Baseline	1.26±0.67	Baseline	Unavailable
			Outcome	5.30±0.60	Outcome	7.21±3.11	Outcome	1.20±0.57	Outcome	Unavailable
Hall, 2006, the UK (26)	Isoflavone	105	Baseline	5.34±0.61	Baseline	5.01±2.62	Baseline	1.21±0.71	Baseline	Unavailable
			Outcome	5.21±0.60	Outcome	5.11±2.58	Outcome	1.20±0.67	Outcome	Unavailable
	Control	105	Baseline	5.32±0.58	Baseline	5.13±2.60	Baseline	1.24±0.65	Baseline	Unavailable
			Outcome	5.27±0.57	Outcome	5.02±3.03	Outcome	1.21±0.75	Outcome	Unavailable

SUPPLEMENTAL TABLE 1 Continued

First author, publication year, country	Group	N	Fasting blood glucose		Fasting blood insulin		HOMA-IR		HbA1c	
Ho, 2007, China (27)	Isoflavone	68	Baseline	5.74±1.79	Baseline	Unavailable	Baseline	Unavailable	Baseline	Unavailable
			Outcome	5.81±1.89	Outcome	Unavailable	Outcome	Unavailable	Outcome	Unavailable
	Control	33	Baseline	5.42±1.25	Baseline	Unavailable	Baseline	Unavailable	Baseline	Unavailable
			Outcome	5.77±1.63	Outcome	Unavailable	Outcome	Unavailable	Outcome	Unavailable
Ho, 2007, China (27)	Isoflavone	68	Baseline	5.44±1.43	Baseline	Unavailable	Baseline	Unavailable	Baseline	Unavailable
			Outcome	5.61±1.59	Outcome	Unavailable	Outcome	Unavailable	Outcome	Unavailable
	Control	34	Baseline	5.42±1.25	Baseline	Unavailable	Baseline	Unavailable	Baseline	Unavailable
			Outcome	5.77±1.63	Outcome	Unavailable	Outcome	Unavailable	Outcome	Unavailable
Irace, 2013, Italy (28)	Isoflavone	10	Baseline	7.18±1.99	Baseline	10.10±3.40	Baseline	3.40±2.20	Baseline	Unavailable
			Outcome	6.69±2.31	Outcome	9.80±3.50	Outcome	2.90±1.50	Outcome	Unavailable
	Control	10	Baseline	6.99±2.39	Baseline	11.30±4.10	Baseline	3.80±2.80	Baseline	Unavailable
			Outcome	6.47±1.71	Outcome	11.20±4.20	Outcome	3.30±1.70	Outcome	Unavailable
Jayagopal, 2002, the UK (29)	Soy protein + isoflavone	32	Baseline	7.29±1.49	Baseline	16.70±8.13	Baseline	5.54±3.40	Baseline	6.83±0.64
			Outcome	7.37±1.63	Outcome	14.50±6.58	Outcome	4.94±2.94	Outcome	6.78±0.61
	Control	32	Baseline	7.23±1.37	Baseline	15.50±7.26	Baseline	5.14±3.04	Baseline	6.82±0.66
			Outcome	7.57±1.93	Outcome	17.30±10.70	Outcome	6.09±4.82	Outcome	6.88±0.59
Jamilian, 2015, Iran (30)	Whole soy	34	Baseline	5.30±0.53	Baseline	16.90±9.50	Baseline	4.10±2.30	Baseline	Unavailable
			Outcome	4.59±0.52	Outcome	16.00±10.10	Outcome	3.30±2.20	Outcome	Unavailable
	Control	34	Baseline	5.11±0.53	Baseline	13.60±5.50	Baseline	3.10±1.40	Baseline	Unavailable
			Outcome	5.18±0.59	Outcome	18.60±12.60	Outcome	4.30±2.80	Outcome	Unavailable
Jamilian,2016, Iran (31)	Isoflavone	35	Baseline	5.07±0.49	Baseline	11.60±5.60	Baseline	2.70±1.40	Baseline	Unavailable
			Outcome	5.16±0.28	Outcome	10.40±2.90	Outcome	2.40±0.80	Outcome	Unavailable
	Control	35	Baseline	5.19±0.34	Baseline	10.10±6.00	Baseline	2.40±1.50	Baseline	Unavailable
			Outcome	5.21±0.39	Outcome	12.90±7.20	Outcome	3.00±1.80	Outcome	Unavailable
Kwak, 2010, Korea (32)	Soy protein	21	Baseline	6.76±0.75	Baseline	11.90±7.47	Baseline	Unavailable	Baseline	6.70±0.64
			Outcome	6.55±1.03	Outcome	17.20±26.99	Outcome	Unavailable	Outcome	6.65±0.64
	Control	21	Baseline	6.41±0.77	Baseline	10.57±2.80	Baseline	Unavailable	Baseline	6.42±0.60
			Outcome	6.35±0.92	Outcome	11.15±4.17	Outcome	Unavailable	Outcome	6.45±0.64
Kwak, 2012, Korea (33)	Soy protein	35	Baseline	5.14±0.71	Baseline	12.20±4.85	Baseline	2.79±1.18	Baseline	Unavailable
			Outcome	4.97±0.76	Outcome	11.10±4.44	Outcome	2.48±1.12	Outcome	Unavailable
	Control	29	Baseline	5.04±0.89	Baseline	12.90±6.09	Baseline	2.96±1.67	Baseline	Unavailable
			Outcome	4.84±0.80	Outcome	13.30±8.45	Outcome	2.93±2.10	Outcome	Unavailable

SUPPLEMENTAL TABLE 1 Continued

**First author, publication year, country**	**Group**	**N**	**Fasting blood glucose**		**Fasting blood insulin**		**HOMA-IR**		**HbA1c**	
Kwak, 2013, Korea (34)	Soy protein	45	Baseline	5.06±0.65	Baseline	8.31±4.56	Baseline	1.88±1.07	Baseline	Unavailable
			Outcome	5.12±0.64	Outcome	7.50±2.30	Outcome	1.71±0.58	Outcome	Unavailable
	Control	46	Baseline	4.99±0.57	Baseline	8.16±3.66	Baseline	1.84±0.90	Baseline	Unavailable
			Outcome	5.09±0.6	Outcome	8.87±4.69	Outcome	2.02±1.04	Outcome	Unavailable
Kim, 2013, Korea (35)	Isoflavone	42	Baseline	5.18±0.42	Baseline	5.30±2.80	Baseline	1.20±0.70	Baseline	Unavailable
			Outcome	5.05±0.51	Outcome	6.10±5.30	Outcome	1.50±1.40	Outcome	Unavailable
	Control	43	Baseline	5.21±0.47	Baseline	5.30±2.50	Baseline	1.20±0.60	Baseline	Unavailable
			Outcome	5.13±0.68	Outcome	5.80±3.30	Outcome	1.40±0.90	Outcome	Unavailable
Kani, 2017, Iran (36)	Whole soy	15	Baseline	6.43±1.16	Baseline	11.30±3.87	Baseline	Unavailable	Baseline	Unavailable
			Outcome	5.78±0.77	Outcome	6.10±3.10	Outcome	Unavailable	Outcome	Unavailable
	Control	15	Baseline	5.84±1.12	Baseline	10.00±4.26	Baseline	Unavailable	Baseline	Unavailable
			Outcome	5.49±0.93	Outcome	8.80±3.10	Outcome	Unavailable	Outcome	Unavailable
Karamali,2018, Iran (37)	Whole soy	30	Baseline	5.00±0.70	Baseline	Unavailable	Baseline	Unavailable	Baseline	Unavailable
			Outcome	4.80±0.50	Outcome	Unavailable	Outcome	Unavailable	Outcome	Unavailable
	Control	30	Baseline	5.10±0.40	Baseline	Unavailable	Baseline	Unavailable	Baseline	Unavailable
			Outcome	5.20±0.40	Outcome	Unavailable	Outcome	Unavailable	Outcome	Unavailable
Konya, 2019, the U.K (38)	Soy protein	13	Baseline	6.39±1.15	Baseline	19.20±9.42	Baseline	5.41±3.14	Baseline	6.22±0.57
			Outcome	6.27±1.20	Outcome	15.75±7.30	Outcome	4.54±2.33	Outcome	6.17±0.48
	Soy protein + isoflavone	13	Baseline	6.69±1.18	Baseline	19.07±12.55	Baseline	5.53±3.22	Baseline	6.42±0.53
			Outcome	7.20±1.78	Outcome	22.70±15.19	Outcome	7.52±5.84	Outcome	6.23±0.58
Lukaczer, 2006 (39)	Soy protein + isoflavone	22	Baseline	5.23±0.75	Baseline	8.56±4.46	Baseline	Unavailable	Baseline	Unavailable
			Outcome	5.03±0.52	Outcome	6.36±3.83	Outcome	Unavailable	Outcome	Unavailable
	Control	20	Baseline	4.97±0.54	Baseline	8.51±4.30	Baseline	Unavailable	Baseline	Unavailable
			Outcome	5.06±0.54	Outcome	9.65±4.16	Outcome	Unavailable	Outcome	Unavailable
Liao, 2007, China (40)	Whole soy	15	Baseline	5.60±0.54	Baseline	Unavailable	Baseline	Unavailable	Baseline	Unavailable
			Outcome	5.44±0.34	Outcome	Unavailable	Outcome	Unavailable	Outcome	Unavailable
	Control	15	Baseline	5.83±0.97	Baseline	Unavailable	Baseline	Unavailable	Baseline	Unavailable
			Outcome	5.39±0.41	Outcome	Unavailable	Outcome	Unavailable	Outcome	Unavailable

SUPPLEMENTAL TABLE 1 Continued

First author, publication year, country	Group	N	Fasting blood glucose		Fasting blood insulin		HOMA-IR		HbA1c	
Liu, 2010, China (41)	Soy protein + isoflavone	60	Baseline	6.40±0.74	Baseline	10.10±5.73	Baseline	2.94±2.12	Baseline	Unavailable
			Outcome	6.30±0.92	Outcome	9.70±5.68	Outcome	2.84±2.45	Outcome	Unavailable
	Isoflavone	60	Baseline	6.40±1.20	Baseline	10.70±6.30	Baseline	3.05±1.92	Baseline	Unavailable
			Outcome	6.20±0.94	Outcome	10.10±5.96	Outcome	2.83±1.79	Outcome	Unavailable
	Control	60	Baseline	6.30±0.89	Baseline	10.30±4.49	Baseline	2.90±1.40	Baseline	Unavailable
			Outcome	6.10±0.74	Outcome	9.40±5.72	Outcome	2.59±1.72	Outcome	Unavailable
Liu, 2014, China (42)	Whole soy	90	Baseline	5.39±0.74	Baseline	Unavailable	Baseline	Unavailable	Baseline	Unavailable
			Outcome	5.40±0.69	Outcome	Unavailable	Outcome	Unavailable	Outcome	Unavailable
	Isoflavone	90	Baseline	5.30±0.82	Baseline	Unavailable	Baseline	Unavailable	Baseline	Unavailable
			Outcome	5.31±0.67	Outcome	Unavailable	Outcome	Unavailable	Outcome	Unavailable
	Control	90	Baseline	5.38±0.63	Baseline	Unavailable	Baseline	Unavailable	Baseline	Unavailable
			Outcome	5.44±0.6	Outcome	Unavailable	Outcome	Unavailable	Outcome	Unavailable
Maleki，2019, Iran (43)	Whole soy	31	Baseline	5.16±0.66	Baseline	12.70±4.40	Baseline	1.63±0.55	Baseline	Unavailable
			Outcome	4.97±0.63	Outcome	9.26±4.42	Outcome	1.18±0.54	Outcome	Unavailable
	Control	31	Baseline	5.18±0.59	Baseline	12.86±2.56	Baseline	1.66±0.32	Baseline	Unavailable
			Outcome	5.09±0.57	Outcome	11.76±4.05	Outcome	1.51±0.52	Outcome	Unavailable
Nikander, 2004, Finland (44)	Isoflavone	28	Baseline	5.10±1.10	Baseline	6.80±2.40	Baseline	Unavailable	Baseline	Unavailable
			Outcome	5.60±2.20	Outcome	7.60±5.30	Outcome	Unavailable	Outcome	Unavailable
	Control	28	Baseline	5.10±0.90	Baseline	7.00±2.10	Baseline	Unavailable	Baseline	Unavailable
			Outcome	5.10±0.90	Outcome	6.70±3.20	Outcome	Unavailable	Outcome	Unavailable
Padhi, 2015, Canada (45)	Whole soy	82	Baseline	5.37±0.45	Baseline	Unavailable	Baseline	Unavailable	Baseline	Unavailable
			Outcome	5.34±0.45	Outcome	Unavailable	Outcome	Unavailable	Outcome	Unavailable
	Control	41	Baseline	5.48±0.36	Baseline	Unavailable	Baseline	Unavailable	Baseline	Unavailable
			Outcome	5.47±0.45	Outcome	Unavailable	Outcome	Unavailable	Outcome	Unavailable
Padhi, 2015, Canada (45)	Whole soy	80	Baseline	5.40±0.54	Baseline	Unavailable	Baseline	Unavailable	Baseline	Unavailable
			Outcome	5.34±0.45	Outcome	Unavailable	Outcome	Unavailable	Outcome	Unavailable
	Control	40	Baseline	5.48±0.36	Baseline	Unavailable	Baseline	Unavailable	Baseline	Unavailable
			Outcome	5.47±0.45	Outcome	Unavailable	Outcome	Unavailable	Outcome	Unavailable

SUPPLEMENTAL TABLE 1 Continued

First author, publication year, country	Group	N	Fasting blood glucose		Fasting blood insulin		HOMA-IR		HbA1c	
Qin, 2013, China (46)	Soy protein + isoflavone	58	Baseline	5.63±0.90	Baseline	5.40±4.57	Baseline	Unavailable	Baseline	Unavailable
			Outcome	5.67±0.95	Outcome	6.83±4.49	Outcome	Unavailable	Outcome	Unavailable
	Soy protein	29	Baseline	5.45±0.78	Baseline	5.63±4.32	Baseline	Unavailable	Baseline	Unavailable
			Outcome	5.39±0.79	Outcome	6.25±3.83	Outcome	Unavailable	Outcome	Unavailable
Qin, 2013, China (46)	Soy protein + isoflavone	60	Baseline	5.50±0.82	Baseline	6.03±5.25	Baseline	Unavailable	Baseline	Unavailable
			Outcome	5.60±0.84	Outcome	7.01±3.85	Outcome	Unavailable	Outcome	Unavailable
	Soy protein	30	Baseline	5.45±0.78	Baseline	5.63±4.32	Baseline	Unavailable	Baseline	Unavailable
			Outcome	5.39±0.79	Outcome	6.25±3.83	Outcome	Unavailable	Outcome	Unavailable
Reverri, 2015, USA (47)	Whole soy	17	Baseline	6.06±0.444	Baseline	12.90±6.50	Baseline	Unavailable	Baseline	Unavailable
			Outcome	0.00±0.00	Outcome	0.08±0.08	Outcome	Unavailable	Outcome	Unavailable
	Control	17	Baseline	6.99±0.62	Baseline	12.70±7.50	Baseline	Unavailable	Baseline	Unavailable
			Outcome	0.00±0.00	Outcome	0.10±0.08	Outcome	Unavailable	Outcome	Unavailable
Sites, 2007, USA (48)	Soy protein + isoflavone	9	Baseline	4.71±0.26	Baseline	17.60±3.10	Baseline	Unavailable	Baseline	Unavailable
			Outcome	-0.02±0.05	Outcome	-1.10±1.60	Outcome	Unavailable	Outcome	Unavailable
	Control	6	Baseline	4.61±0.26	Baseline	15.30±2.40	Baseline	Unavailable	Baseline	Unavailable
			Outcome	0.10±0.14	Outcome	1.90±2.00	Outcome	Unavailable	Outcome	Unavailable
StOnge, 2007, USA (49)	Soy protein	24	Baseline	4.97±0.90	Baseline	9.40±4.41	Baseline	Unavailable	Baseline	Unavailable
			Outcome	4.84±0.49	Outcome	8.00±3.43	Outcome	Unavailable	Outcome	Unavailable
	Control	23	Baseline	4.62±0.53	Baseline	6.90±2.40	Baseline	Unavailable	Baseline	Unavailable
			Outcome	4.63±0.40	Outcome	6.30±1.44	Outcome	Unavailable	Outcome	Unavailable
Santo, 2008, USA (50)	Soy protein + isoflavone	10	Baseline	4.62±0.84	Baseline	Unavailable	Baseline	Unavailable	Baseline	Unavailable
			Outcome	5.21±0.54	Outcome	Unavailable	Outcome	Unavailable	Outcome	Unavailable
	Soy protein	11	Baseline	5.24±0.77	Baseline	Unavailable	Baseline	Unavailable	Baseline	Unavailable
			Outcome	5.25±0.57	Outcome	Unavailable	Outcome	Unavailable	Outcome	Unavailable
	Control	9	Baseline	5.39±0.63	Baseline	Unavailable	Baseline	Unavailable	Baseline	Unavailable
			Outcome	5.36±1.00	Outcome	Unavailable	Outcome	Unavailable	Outcome	Unavailable

SUPPLEMENTAL TABLE 1 Continued

**First author, publication year, country**	**Group**	**N**	**Fasting blood glucose**		**Fasting blood insulin**		**HOMA-IR**		**HbA1c**	
Sathyapalan, 2011, the UK (51)	Soy protein	48	Baseline	5.30±0.21	Baseline	27.50±8.31	Baseline	3.50±0.55	Baseline	Unavailable
			Outcome	5.20±0.28	Outcome	26.90±6.24	Outcome	3.40±0.42	Outcome	Unavailable
	Soy protein + isoflavone	48	Baseline	5.40±1.39	Baseline	27.30±6.24	Baseline	3.50±0.62	Baseline	Unavailable
			Outcome	4.90±0.35	Outcome	20.80±8.31	Outcome	2.60±0.55	Outcome	Unavailable
Squadrito, 2013, Italy (52)	Isoflavone	55	Baseline	7.79±4.33	Baseline	12.7±16.65	Baseline	4.50±8.32	Baseline	Unavailable
			Outcome	6.59±2.94	Outcome	9.30±5.86	Outcome	2.70±1.70	Outcome	Unavailable
	Control	53	Baseline	7.97±5.59	Baseline	12.00±10.59	Baseline	4.30±4.09	Baseline	Unavailable
			Outcome	7.67±10.35	Outcome	11.80±23.77	Outcome	4.10±13.00	Outcome	Unavailable
Sathyapalan, 2016, the UK (53)	Soy protein + isoflavone	60	Baseline	5.20±0.70	Baseline	5.78±3.59	Baseline	1.39±1.03	Baseline	Unavailable
			Outcome	4.40±0.50	Outcome	2.64±1.89	Outcome	0.52±0.40	Outcome	Unavailable
	Soy protein	60	Baseline	5.10±1.60	Baseline	5.65±3.74	Baseline	1.43±1.77	Baseline	Unavailable
			Outcome	5.00±0.90	Outcome	5.82±3.70	Outcome	1.37±1.42	Outcome	Unavailable
Sathyapalan, 2017, the UK (54)	Soy protein + isoflavone	85	Baseline	Unavailable	Baseline	Unavailable	Baseline	Unavailable	Baseline	Unavailable
			Outcome	-1.44±1.61	Outcome	-10.96±13.51	Outcome	-4.42±5.73	Outcome	Unavailable
	Soy protein	86	Baseline	Unavailable	Baseline	Unavailable	Baseline	Unavailable	Baseline	Unavailable
			Outcome	0.59±2.02	Outcome	-0.70±25.78	Outcome	0.78±17.67	Outcome	Unavailable
Sedaghat, 2019, Iran (55)	Whole soy	34	Baseline	9.19±2.28	Baseline	Unavailable	Baseline	Unavailable	Baseline	Unavailable
			Outcome	8.23±2.11	Outcome	Unavailable	Outcome	Unavailable	Outcome	Unavailable
	Control	34	Baseline	9.31±1.79	Baseline	Unavailable	Baseline	Unavailable	Baseline	Unavailable
			Outcome	8.98±1.73	Outcome	Unavailable	Outcome	Unavailable	Outcome	Unavailable
Thirunavukkarasu, 2017, India (56)	Whole soy	25	Baseline	6.35±0.40	Baseline	29.76±3.00	Baseline	8.40±1.10	Baseline	Unavailable
			Outcome	5.92±0.35	Outcome	24.32±2.65	Outcome	6.42±1.00	Outcome	Unavailable
	Control	12	Baseline	6.29±0.46	Baseline	29.52±3.50	Baseline	8.26±1.30	Baseline	Unavailable
			Outcome	6.25±0.42	Outcome	29.08±3.10	Outcome	8.19±1.15	Outcome	Unavailable
Thirunavukkarasu, 2017, India (56)	Whole soy	25	Baseline	6.31±0.33	Baseline	30.72±3.4	Baseline	8.60±1.00	Baseline	Unavailable
			Outcome	5.53±0.41	Outcome	25.20±1.50	Outcome	6.19±0.60	Outcome	Unavailable
	Control	13	Baseline	6.29±0.46	Baseline	29.52±3.52	Baseline	8.26±1.30	Baseline	Unavailable
			Outcome	6.25±0.42	Outcome	29.08±3.10	Outcome	8.19±1.15	Outcome	Unavailable

SUPPLEMENTAL TABLE 1 Continued

First author, publication year, country	Group	N	Fasting blood glucose		Fasting blood insulin		HOMA-IR		HbA1c	
Villa, 2009, Italy (57)	Isoflavone	25	Baseline	5.03±2.87	Baseline	10.17±28.90	Baseline	2.22±5.30	Baseline	Unavailable
			Outcome	4.93±3.22	Outcome	7.24±14.65	Outcome	1.60±3.60	Outcome	Unavailable
	Control	18	Baseline	4.89±2.11	Baseline	9.50±12.35	Baseline	2.01±1.19	Baseline	Unavailable
			Outcome	4.87±1.48	Outcome	9.70±12.52	Outcome	2.10±1.02	Outcome	Unavailable
van Nielen, 2014, Netherlands (58)	Whole soy	15	Baseline	5.60±0.70	Baseline	3.80±1.80	Baseline	1.00±0.50	Baseline	Unavailable
			Outcome	5.40±0.50	Outcome	3.40±1.70	Outcome	0.50±0.20	Outcome	Unavailable
	Control	15	Baseline	5.70±0.60	Baseline	4.00±2.20	Baseline	1.00±0.60	Baseline	Unavailable
			Outcome	5.40±0.40	Outcome	3.00±1.40	Outcome	0.40±0.20	Outcome	Unavailable
Wu, 2005, USA (59)	Whole soy	17	Baseline	Unavailable	Baseline	11.94±7.08	Baseline	Unavailable	Baseline	Unavailable
			Outcome	Unavailable	Outcome	8.66±4.59	Outcome	Unavailable	Outcome	Unavailable
	Control	20	Baseline	Unavailable	Baseline	9.93±5.90	Baseline	Unavailable	Baseline	Unavailable
			Outcome	Unavailable	Outcome	8.66±4.69	Outcome	Unavailable	Outcome	Unavailable
Welty, 2007, Israel (60)	Whole soy	55	Baseline	Unavailable	Baseline	Unavailable	Baseline	Unavailable	Baseline	Unavailable
			Outcome	5.45±0.56	Outcome	Unavailable	Outcome	Unavailable	Outcome	Unavailable
	Control	27	Baseline	Unavailable	Baseline	Unavailable	Baseline	Unavailable	Baseline	Unavailable
			Outcome	5.39±0.50	Outcome	Unavailable	Outcome	Unavailable	Outcome	Unavailable
Ye, 2015, China (61)	Soy protein + isoflavone	55	Baseline	5.70±0.80	Baseline	8.20±9.10	Baseline	2.10±2.50	Baseline	6.40±0.60
			Outcome	5.60±0.80	Outcome	8.30±6.10	Outcome	2.10±1.60	Outcome	6.40±0.60
	Soy protein	27	Baseline	5.80±0.90	Baseline	8.20±10.50	Baseline	2.10±3.70	Baseline	6.40±1.10
			Outcome	5.70±1.10	Outcome	7.90±5.20	Outcome	2.00±1.30	Outcome	6.30±0.70
Ye, 2015, China (61)	Soy protein + isoflavone	56	Baseline	5.80±0.90	Baseline	8.20±5.50	Baseline	2.20±1.50	Baseline	6.40±0.80
			Outcome	5.50±0.90	Outcome	7.50±5.90	Outcome	1.80±1.50	Outcome	6.40±0.80
	Soy protein	27	Baseline	5.80±0.90	Baseline	8.20±10.50	Baseline	2.10±3.70	Baseline	6.40±1.10
			Outcome	5.70±1.10	Outcome	7.90±5.20	Outcome	2.00±1.30	Outcome	6.30±0.70

SUPPLEMENTAL TABLE 2 Characteristics of eligible studies.[1]

First author, publication year, country	Study design	Group	Duration (weeks)	No. of participants (Inter, Ctrl)	Gender	Age	BMI	Health status	Inter. group treatment	Ctrl. group treatment	Extracted Outcomes
Azadbakht, 2006, Iran (1)	R, C	SP, whole soy, control	8	42	Female	NR	-	Postmenopausal, metabolic syndrome.	SP diet (84 mg IF, 15 g SP), soy-nut diet (102 mg IF, 11.3 g SP)	Control diet (0 mg IF, 0 g SP)	FBG, FBI, HOMA-IR
Atteritano, 2007, Italy (2)	R, P	IF, control	104	389	Female	54.5	25.1	Osteopenic, postmenopausal	IF tablets (54 mg IF)	Placebo tablets (0 mg IF)	FBG, FBI, HOMA-IR
Aubertin-Leheudre, 2008, Canada (3)	R, P	IF, control	24	39	Female	57.4	31.9	Obese, postmenopausal	Isoflavone capsules (70 mg IF)	Placebo capsules (0 mg IF)	FBG, FBI, HOMA-IR
Acharjee, 2015, USA (4)	R, C	SP + IF, control	8	11	Female	NR	NR	Postmenopausal, metabolic syndrome, postmenopausal, without metabolic syndrome	Soy nuts (101 mg IF, 25 g SP)	Control (0 mg IF, 0 g SP)	FBI
Amanat,2018, Iran (5)	R, P	IF, control	8	78	Female/ Male	43.6	28.5	Non-alcoholic fatty liver	Genistein capsules (250 mg IF)	Cornstarch capsules (0 mg IF)	FBG, FBI, HOMA-IR
Barsalani, 2013, Canada (6)	R, P	IF, control	24	54	Female	NR	30.0	Healthy, overweight to obese, postmenopausal	IF capsule (70 mg IF) + exercise	Placebo capsules (0 mg IF) + exercise	FBG, FBI, HOMA-IR
Beavers, 2015, USA (7)	R, P	SP + IF, control	12	24	Female/ Male	69.5	36.1	Older, abdominally obesity	SP-based meal replacements (60.135 mg IF, 44 g SP)	Non-SP-based meal replacements (0 mg IF, 0 g SP)	FBG, FBI
Braxas, 2019, Iran (8)	R, P	IF, control	12	54	Female	57.7	31.1	Postmenopausal, type 2 diabetes mellitus	Genistein capsules (54 mg IF)	Placebo capsules (54 mg IF)	FBG, FBI, HOMA-IR
Bakhtiari, 2019, Iran (9)	R, P	Whole soy, SP + IF, control	12	225	Female	64.1	28.3	Older, metabolic syndrome	Roasted soy-nut (117.2 mg IF, 13.8 g SP), textured SP (92.6 mg IF, 18.2 g SP)	No intervention (0 mg IF,0 g SP)	FBG, FBI, HOMA-IR
Colacurci, 2005, Italy (10)	R, P	IF, control	24	57	Female	55.2	25.9	Healthy, postmenopausal	IF tablets (60 mg IF)	Placebo tablets (0 mg IF)	FBG

SUPPLEMENTAL TABLE 2 Continued

First author, publication year, country	Study design	Group	Duration (weeks)	No. of participants (Inter, Ctrl)	Gender	Age	BMI	Health status	Inter. group treatment	Ctrl. group treatment	Extracted Outcomes
Crisafulli, 2005, Italy (11)	R, P	IF, control	24	60	Female	55.5	23.5	Healthy, postmenopausal	Phytoestrogen genistein (54 mg IF)	Placebo (0 mg IF）	FBG, FBI, HOMA-IR
Chan, 2008, China (12)	R, P	IF, control	12	102	Female/ Male	66.3	25.6	Primary or recurrent ischaemic stroke	Soya beans oral (80 mg IF)	Cellulose powdered (0 mg IF)	FBG, FBI
Chang, 2008， Korea (13)	R, P	SP, control	4	20	Female/ Male	55.8	24.8	Type 2 diabetes mellitus	Soybean powder pills (23.9 g SP)	Placebo (0 g SP)	FBG, HOMA-IR
Charles, 2009, USA (14)	R, P	SP + IF, control	12	75	Female	56.6	25.7	Healthy, postmenopausal	Soy powder (160 mg IF, 20g SP)	Whole milk protein (0 mg IF, 0g SP)	FBG, FBI, HOMA-IR
Choquette, 2011, Canada (15)	R, P	IF, control	24	45	Female	58.5	30.1	Postmenopausal, overweight	Soya IF capsules (70 mg IF)	Placebo (0 mg IF）	FBG, FBI, HOMA-IR
Chilibeck, 2013, Canada (16)	R, P	IF, control	104	149	Female	57.7	NR	Postmenopausal	IF therapy (101 mg IF)	Placebo (0 mg IF）	FBI
Duncan, 1999, USA (17)	R, C	SP + IF, SP	13	18	Female	56.9	25.4	Healthy, postmenopausal	Soy protein powders (65 mg IF, 63 g SP), (132 mg IF, 63 g SP)	Soy protein powders (7.1 mg IF, 63 g SP)	FBI
Deibert, 2011, Germany (18)	R, P	SP, control	12	26	Male	55.7	28.1	Previously untrained, middle aged	Resistance training with a soy protein-based supplement (26.7 g SP)	Resistance training without a soy protein-based supplement (0 g SP)	FBG, FBI, HOMA-IR
Garrido, 2006, Chile (19)	R, P	IF, control	12	29	Female	53.5	26.9	Healthy, postmenopausal	Isoflavone capsules (100 mg IF)	Placebo capsules (0 mg IF)	FBI
Gardner, 2007, USA (20)	R, P	Whole soy, SP, control	4	28	Female/ Male	52.0	26.0	Hypercholesterolemic	Whole bean soy milks (125 mg IF, 25 g SP), soy protein isolate milks (39 mg IF, 25 g SP)	Dairy milk (0 mg IF, 0 g SP)	FBG

SUPPLEMENTAL TABLE 2 Continued

First author, publication year, country	Study design	Group	Duration (weeks)	No. of participants (Inter, Ctrl)	Gender	Age	BMI	Health status	Inter. group treatment	Ctrl. group treatment	Extracted Outcomes
Gonzalez, 2007, the UK (21)	R, C	IF, control	12	26	Female	NR	30.9	Postmenopausal, type 2 diabetes	Soy preparation tablets (132 mg IF)	Placebo tablets (132 mg IF)	FBG, FBI, HOMA-IR
Gobert, 2010, Canada (22)	R, C	SP + IF, control	8	29	Female/ Male	60.1	29.4	Type 2 diabetes	Soya pro isolate (88 mg IF, 40 g SP)	Milk pro isolate (0 mg IF, 0 g SP)	FBG, HbA1c
Hermansen, 2001, Denmark (23)	R, C	SP + IF, control	6	20	Female/ Male	61.6	30.2	Type 2 Diabetes	Soy supplement (165 mg IF, 50 g SP)	Casein and cellulos (0 mg IF, 0 g SP)	FBG, FBI, HbA1c
Han, 2002, Brazil (24)	R, P	SP + IF, SP	16	80	Female	48.5	24.9	Healthy, postmenopausal	Isoflavone and SP capsules (100 mg IF, 150.9 g SP)	SP capsules (0 mg IF, 150.9 g SP)	FBG
Hermansen, 2005, Denmark (25)	R, P	SP + IF, control	24	89	NA	59.3	26.0	Hypercholesterolemic	Soy supplement (100 mg IF, 30 g SP)	Casein (0 mg IF, 0 g SP)	FBG, HOMA-IR
Hall, 2006, the UK (26)	R, C	IF, control	8	113	Female	57.7	25.0	Healthy, postmenopausal	Cereal bars (50 mg IF, with genistein: daidzein = 2:1)	Placebo cereal bars (0 mg IF)	FBG, FBI, HOMA-IR
Ho, 2007, China (27)	R, P	IF, control	52	203	Female	54.1	23.9	Postmenopausal	Isoflavone capsules (40 mg IF), (80 mg IF)	Cornstarch capsules (0 mg IF)	FBG
Irace, 2013, Italy (28)	R, P	IF, control	24	20	Female	58.8	31.8	Postmenopausal, metabolic syndrome	IF tables (54 mg IF) + Mediterranean style diet	Placebo tables (0 mg IF) + Mediterranean style diet	FBG, FBI, HOMA-IR
Jayagopal, 2002, the UK (29)	R, C	SP + IF, control	12	32	Female	62.5	32.2	Postmenopausal, type 2 diabetes	Soy preparation (132 mg IF, 30 g SP)	Placebo (0 mg IF, 0 g SP)	FBG, FBI, HOMA-IR, HbA1c
Jamilian, 2015, Iran (30)	R, P	Whole soy, control	6	136	Female/ Male	29.8	28.7	Pregnant women, gestational diabetes mellitus	35% animal pro + 35% soy pro + 30% other plant pro (21 g SP)	70% animal pro + 30% other plant pro (0 g SP)	FBG, FBI

SUPPLEMENTAL TABLE 2 Continued

First author, publication year, country	Study design	Group	Duration (weeks)	No. of participants (Inter, Ctrl)	Gender	Age	BMI	Health status	Inter. group treatment	Ctrl. group treatment	Extracted Outcomes
Jamilian,2016, Iran (31)	R, P	IF, control	12	70	Female	26.7	25.8	Polycystic ovary syndrome	Isoflavones supplements capsules (50 mg IF)	Placebo capsules (0 mg IF)	FBG, FBI, HOMA-IR
Kwak, 2010, Korea (32)	R, P	SP, control	12	42	Female/ Male	57.2	24.5	Prediabetes, newly diagnosed type 2 diabetes mellitus	Black soy peptide tablet (/)	Placebo tablet (/)	FBG, FBI, HbA1c
Kwak, 2012, Korea (33)	R, P	SP, control	12	64	Female/ Male	37.7	27.8	Overweight and obese	Black soy peptide supplementation (/)	Casein placebo (/)	FBG, FBI, HOMA-IR
Kwak, 2013, Korea (34)	R, P	SP, control	8	91	Female/ Male	45.8	25.0	Prehypertension or stage I hypertension	Black soy peptide supplementation (4.5 g peptide)	Placebo (casein) pouches (/)	FBG, FBI, HOMA-IR
Kim, 2013, Korea (35)	R, P	IF, control	12	85	Female	53.6	23.3	Korean postmenopausal women	IF capsule (70 mg IF)	Placebo (0 mg IF）	FBG, FBI, HOMA-IR
Kani, 2017, Iran (36)	R, P	Whole soy, control	8	30	Female/ Male	48.9	NR	Non-alcoholic fatty liver	Low calorie low carbohydrate soy containing diet (102 mg IF, 11.3 g SP)	Low calorie low carbohydrate diet (0 mg IF, 0 g SP)	FBG, FBI
Karamali, 2018, Iran (37)	R, C	Whole soy, control	8	60	Female	25.5	28.3	Polycystic ovary syndrome	Textured SP (98 mg IF, 20.3 g SP)	Animal proteins (0 mg IF, 0 g SP)	FBG
Konya, 2019, the UK (38)	/	Soy protein, Soy protein + IF	8	26	Female/ Male	65.1	31.0	Diet or metformin controlled type 2 diabetes	SP powder bars that were IF free (0 mg IF, 15 g SP)	SP + IF bars (32 mg IF, 15 g SP)	FBG, FBI, HOMA-IR
Lukaczer, 2006, USA (39)	R, P	SP + IF, control	12	42	Female	55.2	32.5	Postmenopausal	A powdered beverage (34 mg IF, 30 g SP)	American heart association step 1 diet (0 mg IF, 0 g SP)	FBG, FBI, HbA1c
Liao, 2007, China (40)	R, P	Whole soy, control	8	30	Female/ Male	33.4	29.8	Overweight	Soy low-calorie diet (45 g SP)	Traditional low-calorie diet (15 g SP)	FBG, HOMA-IR

SUPPLEMENTAL TABLE 2 Continued

First author, publication year, country	Study design	Group	Duration (weeks)	No. of participants (Inter, Ctrl)	Gender	Age	BMI	Health status	Inter. group treatment	Ctrl. group treatment	Extracted Outcomes
Liu, 2010, China (41)	R, P	SP + IF, IF, control	24	183	Female	56.1	24.5	Prediabetes or untreated early diabetes	Supplement preparations (100 mg IF, 15 g SP), (100 mg IF, 0 g SP)	Milk protein (0 mg IF, 0 g SP)	FBG, FBI, HOMA-IR
Liu, 2014, China (42)	R, P	Whole soy, IF, control	24	270	Female	57.9	23.4	Postmenopausal	Soy flour beverage (49.8 mg IF,12.8 g SP, with 23.2mg daidzein and 19.4 mg genistein), low-fat milk powder with daidzein beverage (63 mg IF, 0 g SP)	Low-fat milk powder beverage (0 mg IF, 0 g SP)	FBI, HOMA-IR
Maleki, 2019, Iran (43)	R, P	Whole soy, control	8	62	Female/ Male	45.7	31.1	Non-alcoholic fatty liver	Soy milk (21 mg IF, 6.75 g SP)	Control group (0 mg IF, 0 g SP)	FBG, FBI, HOMA-IR
Nikander, 2004, Finland (44)	R, C	IF, control	12	56	Female	55.0	26.3	Postmenopausal, treated for breast cancer more than 6 months earlier	IF tablets (114 mg IF)	Placebo tablets (0 mg IF)	FBG, FBI
Padhi, 2015, Canada (45)	R, P	Whole soy, control	6	243	Female/ Male	55.0	27.9	Hypercholesterolemic	High-dose soy muffins (106.6 mg IF, 25 g SP), ligh-dose soy muffins (53.3 mg IF, 12.5 g SP)	Wheat muffins (0 mg IF, 0 g SP)	FBG
Qin, 2013, China (46)	R, P	SP + IF, SP + IF, SP	24	177	Female/ Male	53.6	23.7	Hypercholesterolemic	Soy isolated protein powder (40 mg IF, 5 g SP), (80 mg IF, 5 g SP)	Soy isolated protein powder (0 mg IF, 5 g SP)	FBG, FBI
Reverri, 2015, USA (47)	R, C	Whole soy, control	4	17	Female/ Male	56.0	31.2	Cardiometabolic risk	Soy nuts snacks (101 mg IF, 25 g SP)	Control snacks (0 mg IF, 0 g SP)	FBG, FBI
Sites, 2007, USA (48)	R, P	SP + IF, control	12	15	Female	56.1	30.4	Postmenopausal	Daily shake containing soy (160 mg IF, 20 g SP)	Casein isocaloric shake placebo (0 mg IF, 0 g SP)	FBG, FBI
StOnge, 2007, USA (49)	R, P	SP, control	12	48	Female	39.1	30.2	Overweight	SP rich foods (25 g SP)	Control diet (0 g SP)	FBG, FBI
Santo, 2008, USA (50)	R, P	SP + IF, SP, control	4	30	Male	24.2	23.9	Sedentary	SP powder supplement (96.4 mg IF, 25.8 g SP), (1.5 mg IF, 24.9 g SP)	Protein powder supplement (0 mg IF, 0 g SP)	FBG

SUPPLEMENTAL TABLE 2 Continued

First author, publication year, country	Study design	Group	Duration (weeks)	No. of participants (Inter, Ctrl)	Gender	Age	BMI	Health status	Inter. group treatment	Ctrl. group treatment	Extracted Outcomes
Sathyapalan, 2011, the UK (51)	R, C	SP, SP + IF	8	48	Female/ Male	57.2	29.3	Subclinical Hypothyroidism.	Low-dose phytoestrogen preparation (2 mg IF, 30 g SP), high-dose phytoestrogen preparation (16 mg IF, 30 g SP)	/	FBG, FBI, HOMA-IR
Squadrito, 2013, Italy (52)	R, P	IF, control	52	108	Female	55.5	31.8	Postmenopausal, metabolic syndrome	IF tables (54 mg IF) + Mediterranean style diet	Placebo tables (0 mg IF) + Mediterranean style diet	FBG, FBI, HOMA-IR
Sathyapalan, 2016, the UK (53)	R, P	SP + IF, SP	24	120	Female	52.0	25.5	Women during early menopause	15 g soy protein with 66 mg of isoflavones	15 g soy protein alone, isoflavone free	FBG, FBI, HOMA-IR
Sathyapalan, 2017, the UK (54)	R, P	SP + IF, SP	12	171	Male	52.0	31.7	Type 2 Diabetes Mellitus, subclinical Hypogonadism	SPI snack bars (66 mg IF, 15 g SP)	SP snack bars (0 mg IF, 15 g SP)	FBG, FBI, HOMA-IR,
Sedaghat, 2019, Iran (55)	R, P	Whole soy, control	8	68	Female/ Male	50.1	28.6	Type 2 diabetes	60g soy nut in two inter-meals as a part of daily protein (/)	The usual diet of diabetes	FBG
Thirunavukkarasu, 2017, India (56)	R, P	Whole soy, control	12	75	Female	51.70666667	NR	Postmenopausal, pre-diabetes and prehypertension	Soya flour dosa (52.99 mg IF), (86.99 mg IF)	Wheat flour dosa (0 mg IF)	FBG, FBI, HOMA-IR
Villa, 2009, Italy (57)	R, P	IF, control	24	43	Female	54.0	27.2	Postmenopausal	Isoflavone capsules (54 mg IF)	Placebo capsules (0 mg IF)	FBG, FBI, HOMA-IR
van Nielen, 2014, Netherlands (58)	R, P	Whole soy, control	4	30	Female	61.0	NR	Postmenopausal, abdominal obesity	Replace meat with soy meat and soy nuts (48 mg IF, 30 g SP)	Protein of mixed origin (0 mg IF, 0 g SP)	FBG, FBI, HOMA-IR
Wu, 2005, USA (59)	P	Whole soy, control	8	37	Female	58.7	NR	Postmenopausal	The soy diet (50 mg IF, 15 g SP)	The healthy balanced diet (0 mg IF, 0 g SP)	FBI
Welty, 2007, Israel (60)	R, C	Whole soy, control	8	48	Female	53.5	25.4	Postmenopausal, non-hypertensive, postmenopausal, hypertensive	One-half cup of unsalted soy nuts (101 mg IF, 25 g SP)	Placebo (0 mg IF, 0 g SP)	FBG
Ye, 2015, China (61)	R, P	SP + IF, SP	24	82	Female	56.4	24.1	Impaired glucose regulation	Isocaloric powder (50 mg daidzein, 10 g SP), (50 mg geinstein, 10 g SP)	Isocaloric powder (0 g SP)	FBG, FBI, HOMA-IR, HbA1c

[1]C, cross-over; NR, Not report; P, parallel; R, randomized; IF, Isolated isoflavones; SP, Soy protein; SP + IF, Soy protein + isoflavones; FBG, Fasting blood glucose; FBI, Fasting blood insulin.

References

1. Azadbakht L, Kimiagar M, Mehrabi Y, Esmaillzadeh A, Padyab M, Hu FB, et al. Soy inclusion in the diet improves features of the metabolic syndrome: a randomized crossover study in postmenopausal women. The American journal of clinical nutrition. 2007;85(3):735-41.http://doi.org/10.1093/ajcn/85.3.735
2. Atteritano M, Marini H, Minutoli L, Polito F, Bitto A, Altavilla D, et al. Effects of the phytoestrogen genistein on some predictors of cardiovascular risk in osteopenic, postmenopausal women: a two-year randomized, double-blind, placebo-controlled study. The Journal of clinical endocrinology and metabolism. 2007;92(8):3068-75.http://doi.org/10.1210/jc.2006-2295
3. Aubertin-Leheudre M, Lord C, Khalil A, Dionne IJ. Isoflavones and clinical cardiovascular risk factors in obese postmenopausal women: a randomized double-blind placebo-controlled trial. Journal of women's health (2002). 2008;17(8):1363-9.http://doi.org/10.1089/jwh.2008.0836
4. Acharjee S, Zhou JR, Elajami TK, Welty FK. Effect of soy nuts and equol status on blood pressure, lipids and inflammation in postmenopausal women stratified by metabolic syndrome status. Metabolism: clinical and experimental. 2015;64(2):236-43.http://doi.org/10.1016/j.metabol.2014.09.005
5. Amanat S, Eftekhari MH, Fararouei M, Bagheri Lankarani K, Massoumi SJ. Genistein supplementation improves insulin resistance and inflammatory state in non-alcoholic fatty liver patients: A randomized, controlled trial. Clinical nutrition (Edinburgh, Scotland). 2018;37(4):1210-5.http://doi.org/10.1016/j.clnu.2017.05.028
6. Barsalani R, Riesco E, Lavoie JM, Dionne IJ. Effect of exercise training and isoflavones on hepatic steatosis in overweight postmenopausal women. Climacteric : the journal of the International Menopause Society. 2013;16(1):88-95.http://doi.org/10.3109/13697137.2012.662251
7. Beavers KM, Gordon MM, Easter L, Beavers DP, Hairston KG, Nicklas BJ, et al. Effect of protein source during weight loss on body composition, cardiometabolic risk and physical performance in abdominally obese, older adults: a pilot feeding study. The journal of nutrition, health & aging. 2015;19(1):87-95.http://doi.org/10.1007/s12603-015-0438-7
8. Braxas H, Rafraf M, Karimi Hasanabad S, Asghari Jafarabadi M. Effectiveness of Genistein Supplementation on Metabolic Factors and Antioxidant Status in Postmenopausal Women With Type 2 Diabetes Mellitus. Canadian journal of diabetes. 2019;43(7):490-7.http://doi.org/10.1016/j.jcjd.2019.04.007
9. Bakhtiari A, Hajian-Tilaki K, Omidvar S, Nasiri-Amiri F. Clinical and metabolic response to soy administration in older women with metabolic syndrome: a randomized controlled trial. Diabetology & metabolic syndrome. 2019;11:47.http://doi.org/10.1186/s13098-019-0441-y
10. Colacurci N, Chiàntera A, Fornaro F, de Novellis V, Manzella D, Arciello A, et al. Effects of soy isoflavones on endothelial function in healthy postmenopausal women. Menopause (New York, NY). 2005;12(3):299-307.http://doi.org/10.1097/01.gme.0000147017.23173.5b
11. Crisafulli A, Altavilla D, Marini H, Bitto A, Cucinotta D, Frisina N, et al. Effects of the phytoestrogen genistein on cardiovascular risk factors in postmenopausal women. Menopause (New York, NY). 2005;12(2):186-92.http://doi.org/10.1097/00042192-200512020-00013
12. Chan YH, Lau KK, Yiu KH, Li SW, Chan HT, Fong DY, et al. Reduction of C-reactive protein with isoflavone supplement reverses endothelial dysfunction in patients with ischaemic stroke. European heart journal. 2008;29(22):2800-7.http://doi.org/10.1093/eurheartj/ehn409

13. Chang JH, Kim MS, Kim TW, Lee SS. Effects of soybean supplementation on blood glucose, plasma lipid levels, and erythrocyte antioxidant enzyme activity in type 2 diabetes mellitus patients. Nutrition research and practice. 2008;2(3):152-7.http://doi.org/10.4162/nrp.2008.2.3.152
14. Charles C, Yuskavage J, Carlson O, John M, Tagalicud AS, Maggio M, et al. Effects of high-dose isoflavones on metabolic and inflammatory markers in healthy postmenopausal women. Menopause (New York, NY). 2009;16(2):395-400.http://doi.org/10.1097/gme.0b013e3181857979
15. Choquette S, Riesco É, Cormier É, Dion T, Aubertin-Leheudre M, Dionne IJ. Effects of soya isoflavones and exercise on body composition and clinical risk factors of cardiovascular diseases in overweight postmenopausal women: a 6-month double-blind controlled trial. The British journal of nutrition. 2011;105(8):1199-209.http://doi.org/10.1017/s0007114510004897
16. Chilibeck PD, Vatanparast H, Pierson R, Case A, Olatunbosun O, Whiting SJ, et al. Effect of exercise training combined with isoflavone supplementation on bone and lipids in postmenopausal women: a randomized clinical trial. Journal of bone and mineral research : the official journal of the American Society for Bone and Mineral Research. 2013;28(4):780-93.http://doi.org/10.1002/jbmr.1815
17. Duncan AM, Underhill KE, Xu X, Lavalleur J, Phipps WR, Kurzer MS. Modest hormonal effects of soy isoflavones in postmenopausal women. The Journal of clinical endocrinology and metabolism. 1999;84(10):3479-84.http://doi.org/10.1210/jcem.84.10.6067
18. Deibert P, Solleder F, König D, Vitolins MZ, Dickhuth HH, Gollhofer A, et al. Soy protein based supplementation supports metabolic effects of resistance training in previously untrained middle aged males. The aging male : the official journal of the International Society for the Study of the Aging Male. 2011;14(4):273-9.http://doi.org/10.3109/13685538.2011.565091
19. Garrido A, De la Maza MP, Hirsch S, Valladares L. Soy isoflavones affect platelet thromboxane A2 receptor density but not plasma lipids in menopausal women. Maturitas. 2006;54(3):270-6.http://doi.org/10.1016/j.maturitas.2005.12.002
20. Gardner CD, Messina M, Kiazand A, Morris JL, Franke AA. Effect of two types of soy milk and dairy milk on plasma lipids in hypercholesterolemic adults: a randomized trial. Journal of the American College of Nutrition. 2007;26(6):669-77.http://doi.org/10.1080/07315724.2007.10719646
21. González S, Jayagopal V, Kilpatrick ES, Chapman T, Atkin SL. Effects of isoflavone dietary supplementation on cardiovascular risk factors in type 2 diabetes. Diabetes care. 2007;30(7):1871-3.http://doi.org/10.2337/dc06-1814
22. Gobert CP, Pipe EA, Capes SE, Darlington GA, Lampe JW, Duncan AM. Soya protein does not affect glycaemic control in adults with type 2 diabetes. The British journal of nutrition. 2010;103(3):412-21.http://doi.org/10.1017/s0007114509991802
23. Hermansen K, Søndergaard M, Høie L, Carstensen M, Brock B. Beneficial effects of a soy-based dietary supplement on lipid levels and cardiovascular risk markers in type 2 diabetic subjects. Diabetes care. 2001;24(2):228-33.http://doi.org/10.2337/diacare.24.2.228
24. Han KK, Soares JM, Jr., Haidar MA, de Lima GR, Baracat EC. Benefits of soy isoflavone therapeutic regimen on menopausal symptoms. Obstetrics and gynecology. 2002;99(3):389-94.http://doi.org/10.1016/s0029-7844(01)01744-6
25. Hermansen K, Hansen B, Jacobsen R, Clausen P, Dalgaard M, Dinesen B, et al. Effects of soy supplementation on blood lipids and arterial function in hypercholesterolaemic subjects. European journal of clinical nutrition. 2005;59(7):843-50.http://doi.org/10.1038/sj.ejcn.1602151
26. Hall WL, Vafeiadou K, Hallund J, Bugel S, Reimann M, Koebnick C, et al. Soy-isoflavone-enriched foods and markers of lipid and glucose metabolism in postmenopausal women:

interactions with genotype and equol production. The American journal of clinical nutrition. 2006;83(3):592-600.http://doi.org/10.1093/ajcn.83.3.592
27. Ho SC, Chen YM, Ho SS, Woo JL. Soy isoflavone supplementation and fasting serum glucose and lipid profile among postmenopausal Chinese women: a double-blind, randomized, placebo-controlled trial. Menopause (New York, NY). 2007;14(5):905-12.http://doi.org/10.1097/GME.0b013e318032b2d3
28. Irace C, Marini H, Bitto A, Altavilla D, Polito F, Adamo EB, et al. Genistein and endothelial function in postmenopausal women with metabolic syndrome. European journal of clinical investigation. 2013;43(10):1025-31.http://doi.org/10.1111/eci.12139
29. Jayagopal V, Albertazzi P, Kilpatrick ES, Howarth EM, Jennings PE, Hepburn DA, et al. Beneficial effects of soy phytoestrogen intake in postmenopausal women with type 2 diabetes. Diabetes care. 2002;25(10):1709-14.http://doi.org/10.2337/diacare.25.10.1709
30. Jamilian M, Asemi Z. The Effect of Soy Intake on Metabolic Profiles of Women With Gestational Diabetes Mellitus. The Journal of clinical endocrinology and metabolism. 2015;100(12):4654-61.http://doi.org/10.1210/jc.2015-3454
31. Jamilian M, Asemi Z. The Effects of Soy Isoflavones on Metabolic Status of Patients With Polycystic Ovary Syndrome. The Journal of clinical endocrinology and metabolism. 2016;101(9):3386-94.http://doi.org/10.1210/jc.2016-1762
32. Kwak JH, Lee JH, Ahn CW, Park SH, Shim ST, Song YD, et al. Black soy peptide supplementation improves glucose control in subjects with prediabetes and newly diagnosed type 2 diabetes mellitus. Journal of medicinal food. 2010;13(6):1307-12.http://doi.org/10.1089/jmf.2010.1075
33. Kwak JH, Ahn CW, Park SH, Jung SU, Min BJ, Kim OY, et al. Weight reduction effects of a black soy peptide supplement in overweight and obese subjects: double blind, randomized, controlled study. Food & function. 2012;3(10):1019-24.http://doi.org/10.1039/c2fo10244g
34. Kwak JH, Kim M, Lee E, Lee SH, Ahn CW, Lee JH. Effects of black soy peptide supplementation on blood pressure and oxidative stress: a randomized controlled trial. Hypertension research : official journal of the Japanese Society of Hypertension. 2013;36(12):1060-6.http://doi.org/10.1038/hr.2013.79
35. Kim J, Lee H, Lee O, Lee KH, Lee YB, Young KD, et al. Isoflavone supplementation influenced levels of triglyceride and luteunizing hormone in Korean postmenopausal women. Archives of pharmacal research. 2013;36(3):306-13.http://doi.org/10.1007/s12272-013-0059-9
36. Kani AH, Alavian SM, Esmaillzadeh A, Adibi P, Haghighatdoost F, Azadbakht L. Effects of a Low-Calorie, Low-Carbohydrate Soy Containing Diet on Systemic Inflammation Among Patients with Nonalcoholic Fatty Liver Disease: A Parallel Randomized Clinical Trial. Hormone and metabolic research = Hormon- und Stoffwechselforschung = Hormones et metabolisme. 2017;49(9):687-92.http://doi.org/10.1055/s-0042-118707
37. Karamali M, Kashanian M, Alaeinasab S, Asemi Z. The effect of dietary soy intake on weight loss, glycaemic control, lipid profiles and biomarkers of inflammation and oxidative stress in women with polycystic ovary syndrome: a randomised clinical trial. Journal of human nutrition and dietetics : the official journal of the British Dietetic Association. 2018;31(4):533-43.http://doi.org/10.1111/jhn.12545
38. Konya J, Sathyapalan T, Kilpatrick ES, Atkin SL. The Effects of Soy Protein and Cocoa With or Without Isoflavones on Glycemic Control in Type 2 Diabetes. A Double-Blind, Randomized, Placebo-Controlled Study. Frontiers in endocrinology. 2019;10:296.http://doi.org/10.3389/fendo.2019.00296

39. Lukaczer D, Liska DJ, Lerman RH, Darland G, Schiltz B, Tripp M, et al. Effect of a low glycemic index diet with soy protein and phytosterols on CVD risk factors in postmenopausal women. Nutrition (Burbank, Los Angeles County, Calif). 2006;22(2):104-13.http://doi.org/10.1016/j.nut.2005.05.007
40. Liao FH, Shieh MJ, Yang SC, Lin SH, Chien YW. Effectiveness of a soy-based compared with a traditional low-calorie diet on weight loss and lipid levels in overweight adults. Nutrition (Burbank, Los Angeles County, Calif). 2007;23(7-8):551-6.http://doi.org/10.1016/j.nut.2007.05.003
41. Liu ZM, Chen YM, Ho SC, Ho YP, Woo J. Effects of soy protein and isoflavones on glycemic control and insulin sensitivity: a 6-mo double-blind, randomized, placebo-controlled trial in postmenopausal Chinese women with prediabetes or untreated early diabetes. The American journal of clinical nutrition. 2010;91(5):1394-401.http://doi.org/10.3945/ajcn.2009.28813
42. Liu ZM, Ho SC, Chen YM, Ho S, To K, Tomlinson B, et al. Whole soy, but not purified daidzein, had a favorable effect on improvement of cardiovascular risks: a 6-month randomized, double-blind, and placebo-controlled trial in equol-producing postmenopausal women. Molecular nutrition & food research. 2014;58(4):709-17.http://doi.org/10.1002/mnfr.201300499
43. Maleki Z, Jazayeri S, Eslami O, Shidfar F, Hosseini AF, Agah S, et al. Effect of soy milk consumption on glycemic status, blood pressure, fibrinogen and malondialdehyde in patients with non-alcoholic fatty liver disease: a randomized controlled trial. Complementary therapies in medicine. 2019;44:44-50.http://doi.org/10.1016/j.ctim.2019.02.020
44. Nikander E, Tiitinen A, Laitinen K, Tikkanen M, Ylikorkala O. Effects of isolated isoflavonoids on lipids, lipoproteins, insulin sensitivity, and ghrelin in postmenopausal women. The Journal of clinical endocrinology and metabolism. 2004;89(7):3567-72.http://doi.org/10.1210/jc.2003-032229
45. Padhi EM, Blewett HJ, Duncan AM, Guzman RP, Hawke A, Seetharaman K, et al. Whole Soy Flour Incorporated into a Muffin and Consumed at 2 Doses of Soy Protein Does Not Lower LDL Cholesterol in a Randomized, Double-Blind Controlled Trial of Hypercholesterolemic Adults. The Journal of nutrition. 2015;145(12):2665-74.http://doi.org/10.3945/jn.115.219873
46. Qin Y, Shu F, Zeng Y, Meng X, Wang B, Diao L, et al. Daidzein supplementation decreases serum triglyceride and uric acid concentrations in hypercholesterolemic adults with the effect on triglycerides being greater in those with the GA compared with the GG genotype of ESR-β RsaI. The Journal of nutrition. 2014;144(1):49-54.http://doi.org/10.3945/jn.113.182725
47. Reverri EJ, LaSalle CD, Franke AA, Steinberg FM. Soy provides modest benefits on endothelial function without affecting inflammatory biomarkers in adults at cardiometabolic risk. Molecular nutrition & food research. 2015;59(2):323-33.http://doi.org/10.1002/mnfr.201400270
48. Sites CK, Cooper BC, Toth MJ, Gastaldelli A, Arabshahi A, Barnes S. Effect of a daily supplement of soy protein on body composition and insulin secretion in postmenopausal women. Fertility and sterility. 2007;88(6):1609-17.http://doi.org/10.1016/j.fertnstert.2007.01.061
49. St-Onge MP, Claps N, Wolper C, Heymsfield SB. Supplementation with soy-protein-rich foods does not enhance weight loss. Journal of the American Dietetic Association. 2007;107(3):500-5.http://doi.org/10.1016/j.jada.2006.12.002
50. Santo AS, Cunningham AM, Alhassan S, Browne RW, Burton H, Leddy JJ, et al. NMR analysis of lipoprotein particle size does not increase sensitivity to the effect of soy protein on CVD risk when compared with the traditional lipid profile. Applied physiology, nutrition, and metabolism = Physiologie appliquee, nutrition et metabolisme. 2008;33(3):489-500.http://doi.org/10.1139/h08-023
51. Sathyapalan T, Manuchehri AM, Thatcher NJ, Rigby AS, Chapman T, Kilpatrick ES, et al. The effect of soy phytoestrogen supplementation on thyroid status and cardiovascular risk markers in

patients with subclinical hypothyroidism: a randomized, double-blind, crossover study. The Journal of clinical endocrinology and metabolism. 2011;96(5):1442-9.http://doi.org/10.1210/jc.2010-2255
52. Squadrito F, Marini H, Bitto A, Altavilla D, Polito F, Adamo EB, et al. Genistein in the metabolic syndrome: results of a randomized clinical trial. The Journal of clinical endocrinology and metabolism. 2013;98(8):3366-74.http://doi.org/10.1210/jc.2013-1180
53. Sathyapalan T, Aye M, Rigby AS, Fraser WD, Thatcher NJ, Kilpatrick ES, et al. Soy Reduces Bone Turnover Markers in Women During Early Menopause: A Randomized Controlled Trial. Journal of bone and mineral research : the official journal of the American Society for Bone and Mineral Research. 2017;32(1):157-64.http://doi.org/10.1002/jbmr.2927
54. Sathyapalan T, Rigby AS, Bhasin S, Thatcher NJ, Kilpatrick ES, Atkin SL. Effect of Soy in Men With Type 2 Diabetes Mellitus and Subclinical Hypogonadism: A Randomized Controlled Study. The Journal of clinical endocrinology and metabolism. 2017;102(2):425-33.http://doi.org/10.1210/jc.2016-2875
55. Sedaghat A, Shahbazian H, Rezazadeh A, Haidari F, Jahanshahi A, Mahmoud Latifi S, et al. The effect of soy nut on serum total antioxidant, endothelial function and cardiovascular risk factors in patients with type 2 diabetes. Diabetes & metabolic syndrome. 2019;13(2):1387-91.http://doi.org/10.1016/j.dsx.2019.01.057
56. Thirunavukkarasu D. Effect of Soy Flour Intake on Systemic Blood Pressure and Glycemic Control in Post-Menopausal Women with Pre-diabetes and Prehypertension. INTERNATIONAL JOURNAL OF PHARMACEUTICAL EDUCATION AND RESEARCH (IJPER). 2020;51:349.http://doi.org/10.5530/ijper.51.2.42
57. Villa P, Costantini B, Suriano R, Perri C, Macrì F, Ricciardi L, et al. The differential effect of the phytoestrogen genistein on cardiovascular risk factors in postmenopausal women: relationship with the metabolic status. The Journal of clinical endocrinology and metabolism. 2009;94(2):552-8.http://doi.org/10.1210/jc.2008-0735
58. van Nielen M, Feskens EJ, Rietman A, Siebelink E, Mensink M. Partly replacing meat protein with soy protein alters insulin resistance and blood lipids in postmenopausal women with abdominal obesity. The Journal of nutrition. 2014;144(9):1423-9.http://doi.org/10.3945/jn.114.193706
59. Wu AH, Stanczyk FZ, Martinez C, Tseng CC, Hendrich S, Murphy P, et al. A controlled 2-mo dietary fat reduction and soy food supplementation study in postmenopausal women. The American journal of clinical nutrition. 2005;81(5):1133-41.http://doi.org/10.1093/ajcn/81.5.1133
60. Welty FK, Lee KS, Lew NS, Zhou JR. Effect of soy nuts on blood pressure and lipid levels in hypertensive, prehypertensive, and normotensive postmenopausal women. Archives of internal medicine. 2007;167(10):1060-7.http://doi.org/10.1001/archinte.167.10.1060
61. Ye YB, Chen AL, Lu W, Zhuo SY, Liu J, Guan JH, et al. Daidzein and genistein fail to improve glycemic control and insulin sensitivity in Chinese women with impaired glucose regulation: A double-blind, randomized, placebo-controlled trial. Molecular nutrition & food research. 2015;59(2):240-9.http://doi.org/10.1002/mnfr.201400390

SUPPLEMENTAL FIGURE 1 Box plots showing the distribution of the mean study duration (weeks) of the trials across the available direct comparisons.[1]

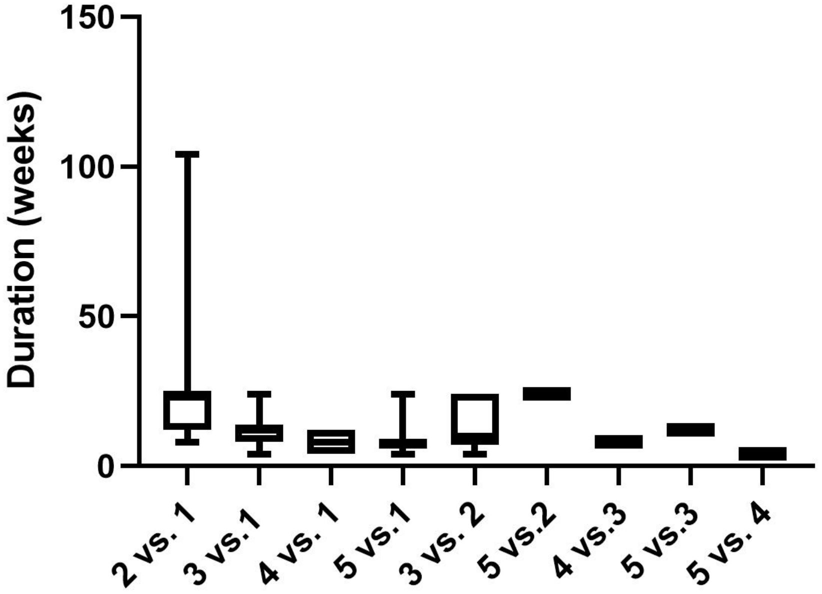

[1]1=Control, 2= Isolated isoflavones, 3= Soy protein + isoflavones, 4= Soy protein, 5= Whole soy

SUPPLEMENTAL FIGURE 2 Box plots showing the distribution of the sample size of the trials across the available direct comparisons.[1]

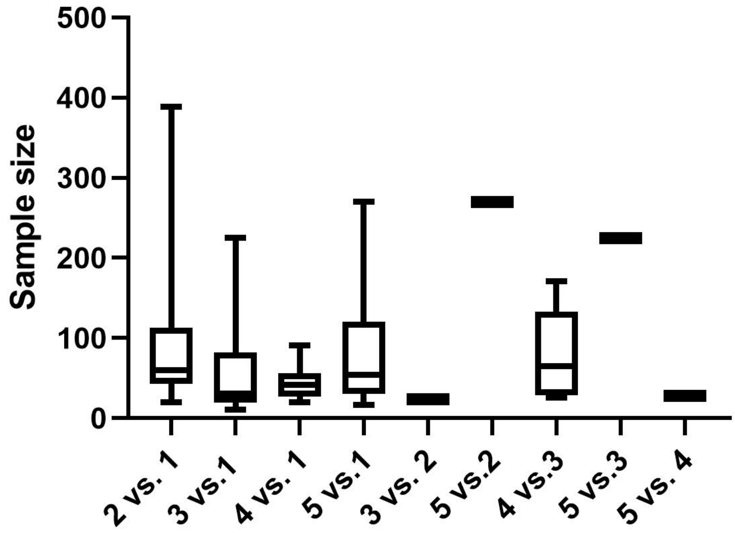

[1]1=Control, 2= Isolated isoflavones, 3= Soy protein + isoflavones, 4= Soy protein, 5= Whole soy

SUPPLEMENTAL FIGURE 3 Box plots showing the distribution of the mean BMI of the trials across the available direct comparisons.[1]

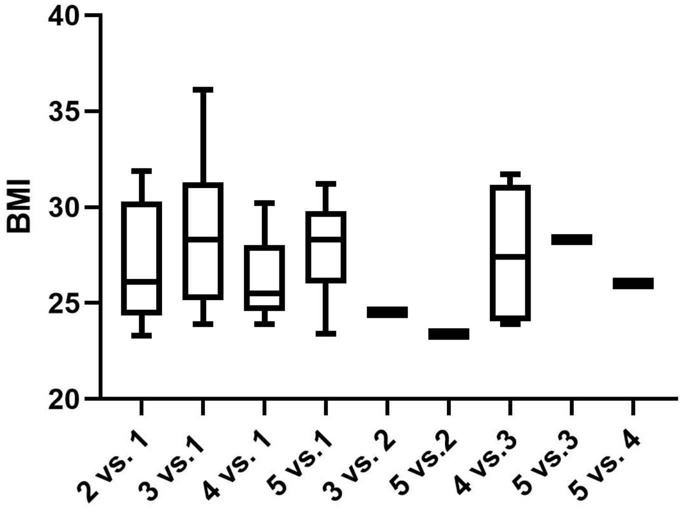

[1]1=Control, 2= Isolated isoflavones, 3= Soy protein + isoflavones, 4= Soy protein, 5= Whole soy

SUPPLEMENTAL FIGURE 4 Box plots showing the distribution of the mean age of the trials across the available direct comparisons.[1]

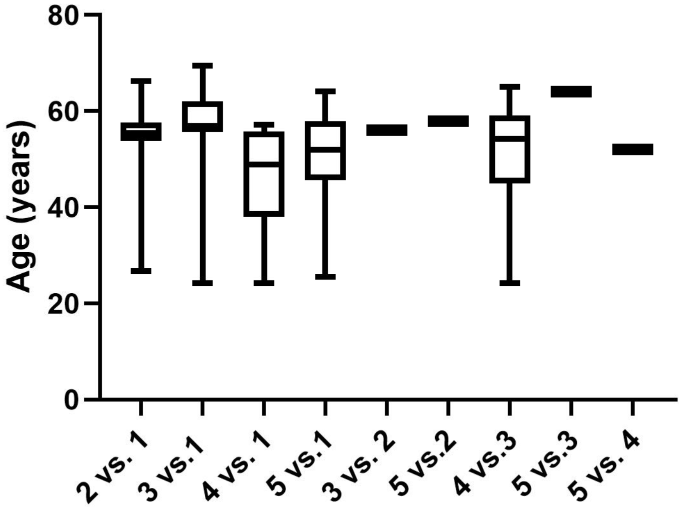

[1]1=Control, 2= Isolated isoflavones, 3= Soy protein + isoflavones, 4= Soy protein, 5= Whole soy

SUPPLEMENTAL TABLE 3 Contribution of direct evidence to the network for blood glucose.

Comparisons	No. of comparisons	Contribution to the network (%)
Control vs Isoflavones	22	20.0
Control vs Soy protein + isoflavones	12	20.4
Control vs Soy protein	7	20.7
Control vs Whole soy	20	17.1
Isoflavones vs Soy protein + isoflavones	1	3.2
Isoflavones vs Soy protein	11	9.3
Soy protein + isoflavones vs Soy protein	11	4.5
Soy protein + isoflavones vs Whole soy	1	3.2
Soy protein vs Whole soy	2	1.7

SUPPLEMENTAL TABLE 4 Contribution of direct evidence to the network for insulin.

Comparisons	No. of comparisons	Contribution to the network (%)
Control vs Isoflavones	17	25.9
Control vs Soy protein + isoflavones	9	14.0
Control vs Soy protein	5	1.9
Control vs Whole soy	11	22.5
Isoflavones vs Soy protein + isoflavones	2	3.3
Isoflavones vs Soy protein	1	14.1
Soy protein + isoflavones vs Soy protein	11	14.3
Soy protein + isoflavones vs Whole soy	1	4.0

SUPPLEMENTAL TABLE 5 Contribution of direct evidence to the network for HOMA-IR.

Comparisons	No. of comparisons	Contribution to the network (%)
Control vs Isoflavones	13	21.4
Control vs Soy protein + isoflavones	7	8.1
Control vs Soy protein	5	26.0
Control vs Whole soy	10	5.6
Isoflavones vs Soy protein + isoflavones	2	2.7
Isoflavones vs Soy protein	1	0.6
Soy protein + isoflavones vs Soy protein	6	8.3
Soy protein + isoflavones vs Whole soy	1	5.5
Soy protein vs Whole soy	1	21.7

SUPPLEMENTAL TABLE 6 Contribution of direct evidence to the network for HbA1c.

Comparisons	No. of comparisons	Contribution to the network (%)
Control vs Isoflavones	1	22.6
Control vs Soy protein + isoflavones	3	25.4
Control vs Soy protein	1	10.5
Control vs Whole soy	1	22.6
Soy protein + isoflavonesvs Soy protein	3	18.9

SUPPLEMENTAL TABLE 7 Assessment of risk of bias of the studies included in the meta-analysis.

First author, publication year, country	Random sequence generation	Allocation concealment	Blinding of participants and personnel	Blinding of Outcome assessment	Incomplete data Outcome	Selective reporting	Other bias
Azadbakht, 2006,Iran (1)	low	unclear	high	unclear	low	low	low
Atteritano, 2007, Italy (2)	low	low	low	unclear	low	low	low
Aubertin-Leheudre, 2008, Canada (3)	low	unclear	low	low	low	unclear	unclear
Acharjee, 2015, USA (4)	low	unclear	unclear	unclear	low	low	low
Amanat,2018, Iran (5)	low	unclear	low	low	low	low	unclear
Barsalani, 2013, Canada (6)	low	low	low	low	unclear	low	unclear
Beavers, 2015, USA (7)	unclear	low	high	unclear	low	low	unclear
Braxas,2019, Iran (8)	low	low	low	low	low	low	low
Bakhtiari, 2019, Iran (9)	low	unclear	high	high	low	low	high
Colacurci, 2005, Italy (10)	unclear	low	unclear	unclear	low	low	unclear
Crisafulli, 2005, Italy (11)	unclear	low	low	low	unclear	low	low
Chan, 2008, China (12)	low	low	low	low	low	low	low
Chang, 2008， Korea (13)	unclear	high	unclear	unclear	low	low	unclear
Charles, 2009, USA (14)	low	low	low	unclear	low	low	low
Choquette, 2010, Canada (15)	unclear	low	low	unclear	low	low	low
Chilibeck, 2013, Canada (16)	low	low	low	low	unclear	low	low
Duncan, 1999, USA (17)	low	unclear	low	unclear	unclear	low	low
Deibert, 2011, Germany (18)	low	unclear	high	unclear	low	low	low
Garrido, 2006, Chile (19)	unclear	low	low	low	unclear	low	low
Gardner, 2007, USA (20)	low	low	low	low	low	unclear	unclear
Gonzalez, 2007, the UK(21)	low	low	low	low	low	low	low
Gobert, 2010, Canada (22)	low	low	low	unclear	low	low	low
Hermansen, 2001, DeUnavailableark (23)	low	low	low	unclear	low	low	low
Han, 2002, Brazil (24)	low	low	low	low	unclear	low	low
Hermansen, 2005, DeUnavailableark (25)	low	low	low	low	unclear	low	low
Hall, 2006, the UK (26)	unclear	low	low	unclear	low	low	low
Ho, 2007, China (27)	low	low	low	low	low	low	low
Irace, 2013, Italy (28)	low	low	low	low	unclear	low	low
Jayagopal, 2002, the UK (29)	low	low	low	unclear	low	low	low
Jamilian, 2015, Iran (30)	low	low	low	low	low	low	low
Jamilian,2016, Iran (31)	low	low	low	low	unclear	low	low
Kwak, 2010, Korea (32)	low	unclear	low	unclear	low	low	unclear
Kwak, 2012, Korea (33)	low	unclear	unclear	high	low	low	unclear
Kwak, 2013, Korea (34)	low	unlear	low	unclear	low	low	low

SUPPLEMENTAL TABLE 7 Continued

First author, publication year, country	Random sequence generation	Allocation concealment	Blinding of participants and personnel	Blinding of Outcome assessment	Incomplete data Outcome	Selective reporting	Other bias
Kim, 2013, Korea (35)	low	low	low	unclear	low	low	low
Kani, 2017, Iran (36)	low	low	high	low	low	low	low
Karamali,2018, Iran (37)	low	unclear	unclear	high	low	low	low
Konya, 2019, the U.K (38)	low	unclear	low	unclear	low	low	unclear
Lukaczer, 2006 (39)	low	unclear	high	unclear	low	low	unclear
Liao, 2007, China (40)	low	unclear	high	unclear	low	low	unclear
Liu, 2010, China (41)	low	low	low	low	low	low	unclear
Liu, 2014, China (42)	low	unlear	low	unclear	low	low	unclear
Maleki，2019, Iran (43)	low	unclear	high	unclear	low	low	low
Nikander, 2004, Finland (44)	low	unclear	low	unclear	low	low	unclear
Padhi, 2015, Canada (45)	low	low	low	low	low	low	unclear
Qin, 2013, China (46)	low	low	low	unclear	low	low	unclear
Reverri, 2015, USA (47)	low	unclear	high	unclear	low	low	low
Sites, 2007, USA (48)	low	unclear	low	unclear	low	low	unclear
StOnge, 2007, USA (49)	low	unclear	high	unclear	low	low	unclear
Santo, 2008, USA (50)	low	unclear	low	unclear	low	low	unclear
Sathyapalan, 2011, the UK (51)	low	low	low	unclear	low	low	unclear
Squadrito, 2013, Italy (52)	low	unclear	low	unclear	low	low	low
Sathyapalan, 2016, the UK (53)	low	low	low	low	low	low	low
Sathyapalan, 2017, the UK (54)	low	unclear	low	unclear	low	low	low
Sedaghat，2019, Iran (55)	low	unclear	high	unclear	low	low	low
Thirunavukkarasu, 2017, India (56)	low	unclear	high	high	low	low	high
Villa, 2009, Italy (57)	low	unclear	unclear	unclear	low	low	low
van Nielen, 2014, Netherlands (58)	low	low	high	unclear	low	low	low
Wu, 2005, USA(59)	low	low	low	low	low	low	low
Welty, 2007, Israel (60)	low	unclear	high	unclear	low	low	low
Ye, 2015, China (61)	low	low	low	low	low	low	low

SUPPLEMENTAL FIGURE 5 Risk of bias graph

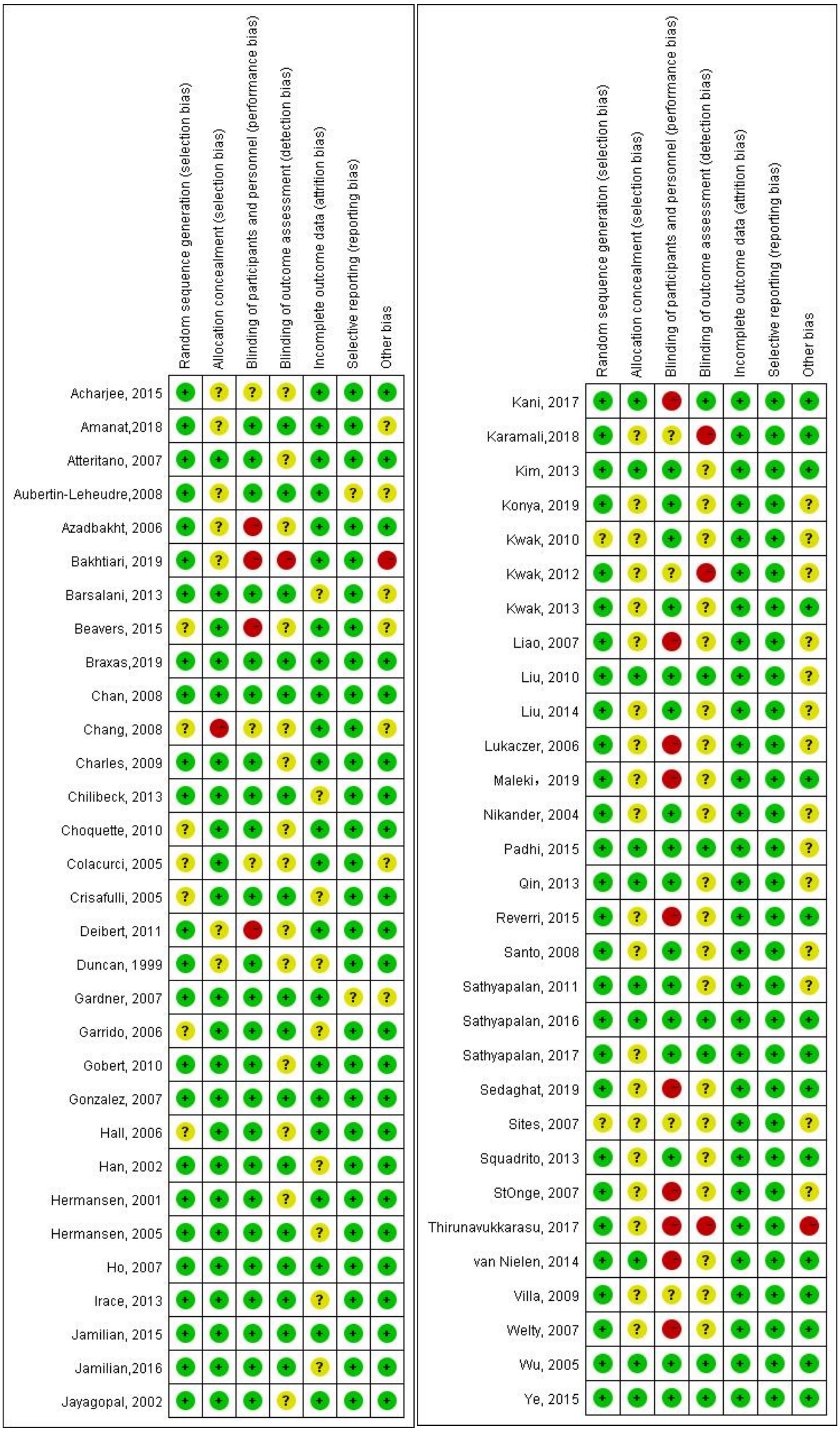

SUPPLEMENTAL FIGURE 6 Risk of bias summary

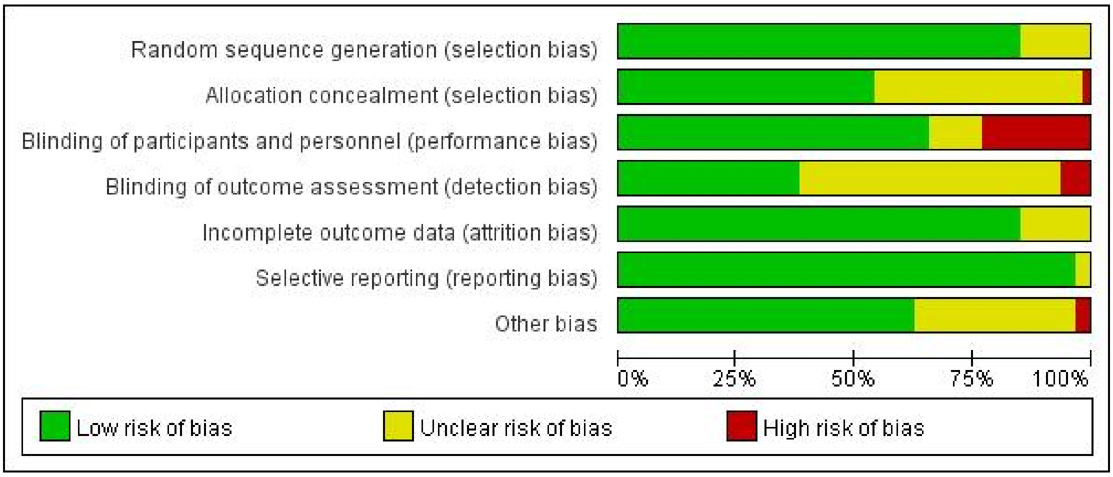

SUPPLEMENTAL TABLE 8 Detailed ranking results of the comparative effects of different types of soy products on the control of blood glucose. The larger the SUCRA value, the better the treatment. SUCRA, surface under the cumulative ranking curve.

Treatments	SUCRA (%)	MeanRank
Control	30.1	3.8
Isoflavones	79.1	1.8
Soy protein + isoflavones	44.8	3.2
Soy protein	5.1	4.8
Whole soy	91.0	1.4

SUPPLEMENTAL TABLE 9 Detailed ranking results of the comparative effects of different types of soy products on the control of insulin. The larger the SUCRA value, the better the treatment. SUCRA, surface under the cumulative ranking curve.

Treatments	SUCRA (%)	MeanRank
Control	24.5	4.0
Isoflavones	75.0	2.0
Soy protein + isoflavones	55.0	2.8
Soy protein	8.4	4.7
Whole soy	87.0	1.5

SUPPLEMENTAL TABLE 10 Detailed ranking results of the comparative effects of different types of soy products on the control of HOMA-IR. The larger the SUCRA value, the better the treatment. SUCRA, surface under the cumulative ranking curve.

Treatments	SUCRA (%)	MeanRank
Control	7.3	4.7
Isoflavones	43.2	3.3
Soy protein + isoflavones	82.8	1.7
Soy protein	33.4	3.7
Whole soy	83.3	1.7

SUPPLEMENTAL TABLE 11 Comparative effects of different types of soy products on the control of HOMA-IR.[1]

	Control	Whole soy	Soy protein	Soy protein + isoflavones	Isoflavones
Control	**-**	**-0.51 (-0.82,-0.19)**	-0.16 (-0.55,0.23)	**-0.50 (-0.85,-0.14)**	-0.22 (-0.54,0.10)
Whole soy		-	0.35 (-0.12,0.81)	0.01 (-0.44,0.46)	0.29 (-0.16,0.73)
Soy protein			-	-0.34 (-0.73,0.06)	-0.06 (-0.55,0.43)
Soy protein + isoflavones				-	0.28 (-0.18,0.73)
Isoflavones					-

[1]Mean differences with 95% confidence intervals (column vs row). Mean differences lower than 0 indicate that the treatments specified in the row is more efficacious than those in the column.

SUPPLEMENTAL TABLE 12 Detailed ranking results of the comparative effects of different types of soy products on the control of HbA1c. The larger the SUCRA value, the better the treatment. SUCRA, surface under the cumulative ranking curve.

Treatments	SUCRA (%)	MeanRank
Control	44.6	3.2
Isoflavones	45.4	3.2
Soy protein + isoflavones	51.8	2.9
Soy protein	60.4	2.6
Whole soy	47.7	3.1

SUPPLEMENTAL TABLE 13 Comparative effects of different types of soy products on the control of HbA1c.[1]

	Control	Whole soy	Soy protein	Soy protein + isoflavones	Isoflavones
Control	-	0.00 (-0.71,0.71)	-0.04 (-0.28,0.19)	-0.02 (-0.21,0.17)	0.00 (-0.23,0.23)
Whole soy		-	-0.04 (-0.79,0.70)	-0.02 (-0.75,0.71)	0.00 (-0.74,0.74)
Soy protein			-	0.03 (-0.16,0.21)	0.04 (-0.29,0.37)
Soy protein + isoflavones				-	0.02 (-0.28,0.32)
Isoflavones					-

[1]Mean differences with 95% confidence intervals (column vs row). Mean differences lower than 0 indicate that the treatments specified in the row is more efficacious than those in the column.

SUPPLEMENTAL TABLE 14 Node-splitting method for assessment of inconsistency for blood glucose.[1]

Comparisons	Direct		Indirect		Difference		P^a	tau
	Coefficient	SE	Coefficient	SE	Coefficient	SE		
Control vs Isoflavones	-.0950702	.0736907	-.2172943	.2785074	.1222241	.2872132	0.670	.1067114
Control vs Soy protein + isoflavones	-.0762301	.1007879	-.0708743	.1907504	-.0053558	.2164139	0.980	.1064139
Control vs Soy protein	.0058032	.1845038	-.1526078	.1230586	.1584110	.2203963	0.472	.0985401
Control vs Whole soy	-.0783993	.0541382	.0065332	.2490471	-.0849325	.2545809	0.739	.1074714
Isoflavones vs Whole soy	.0900138	.1457891	-.0022264	.1018552	.0922402	.1779438	0.604	.1047759
Soy protein + isoflavones vs Soy protein	-.0281393	.0941686	-.0227831	.1940834	-.0053562	.2164145	0.980	.1064146
Soy protein vs Whole soy	.1221287	.1828062	-.0276769	.1434211	.1498057	.2293466	0.514	.106273

[1]*P* for assessment of inconsistency. $P < 0.05$ was considered to indicate a significant inconsistency existed between direct and indirect evidence.

SUPPLEMENTAL TABLE 15 Node-splitting method for assessment of inconsistency for insulin.[1]

Comparisons	Direct		Indirect		Difference		P^a	tau
	Coefficient	SE	Coefficient	SE	Coefficient	SE		
Control vs Isoflavones	-.7104875	.4381672	-6.2355630	2.1595490	5.5250750	2.203819	0.012	1.412964
Control vs Soy protein + isoflavones	-.7788280	.6704424	.1924459	1.1033420	-.9712739	1.292146	0.452	1.591263
Control vs Soy protein	1.1724600	1.0202950	-.0891388	.8988610	1.2615990	1.360447	0.354	1.582009
Control vs Whole soy	-1.343569	.5684180	2.2774460	3.6571130	-3.6210150	3.704694	0.328	1.590985
Isoflavones vs Soy protein + isoflavones	1.0104670	1.2702900	.1436855	.8323661	.8667811	1.516971	0.568	1.608502
Isoflavones vs Soy protein	5.3089220	1.4944730	.3050768	.7811678	5.0038450	1.686144	0.003	1.341859
Soy protein + isoflavones vs Soy protein	.8639073	.5992019	1.6302360	1.4002420	-.7663290	1.519933	0.614	1.605375
Soy protein + isoflavones vs Whole soy	-.0335204	1.9864810	-.8724428	.8542227	.8389224	2.162419	0.698	1.612501

[1]*P* for assessment of inconsistency. $P < 0.05$ was considered to indicate a significant inconsistency existed between direct and indirect evidence.

SUPPLEMENTAL TABLE 16 Node-splitting method for assessment of inconsistency for HOMA-IR.[1]

Comparisons	Direct		Indirect		Difference		P^a	tau
	Coefficient	SE	Coefficient	SE	Coefficient	SE		
Control vs Isoflavones	.0073465	.2293987	-1.1574380	1.1199620	1.1647850	1.1425380	0.308	.4272879
Control vs Soy protein + isoflavones	-.2818420	.2625147	-.4854132	.3478234	.2035712	.4357562	0.640	.4339649
Control vs Soy protein	-.2267481	.2708600	-.0647240	.3601221	-.1620241	.4548219	0.722	.4348813
Control vs Whole soy	-.6660583	.1837763	.0334917	.7438286	-.6995501	.7670165	0.362	.4360534
Isoflavones vs Soy protein + isoflavones	-.0339457	.5855428	-.4138210	.3451800	.3798753	.6804313	0.577	.437444
Soy protein + isoflavones vs Soy protein	.2873501	.2899849	.0421535	.3536548	.2451966	.4619471	0.596	.4294743
Soy protein + isoflavones vs Whole soy	-.1068975	.5433558	-.3241633	.2977553	.2172658	.6195356	0.726	.4452882
Soy protein vs Whole soy	-.3000000	.4488153	-.5439728	.3164968	.2439728	.5491862	0.657	.4468143

[1]*P* for assessment of inconsistency. *P* <0.05 was considered to indicate a significant inconsistency existed between direct and indirect evidence.

SUPPLEMENTAL TABLE 17 Node-splitting method for assessment of inconsistency for HbA1c.[1]

Comparisons	Direct		Indirect		Difference		P^a	tau
	Coefficient	SE	Coefficient	SE	Coefficient	SE		
Control vs Isoflavones								
Control vs Soy protein + isoflavones	-.0950775	.1102503	.2905653	.2220203	-.3856428	.2478874	0.120	6.24e-08
Control vs Soy protein	.2000003	.1975083	-.185643	.1497955	.3856432	.2478875	0.120	4.01e-07
Control vs Whole soy								
Soy protein + isoflavones vs Soy protein	-.0905654	.1014079	.2950778	.226196	-.3856432	.2478875	0.120	1.02e-06

[1]*P* for assessment of inconsistency. *P* <0.05 was considered to indicate a significant inconsistency existed between direct and indirect evidence.

SUPPLEMENTAL FIGURE 7 Evaluation of inconsistency by using loop-specific approach for blood glucose. IFs are calculated as the absolute difference between direct and indirect estimates and therefore confidence intervals are truncated to 0. Loops that their lower CI limit does not reach the 0 line are considered to present statistically significant inconsistency. IF, inconsistency factor.

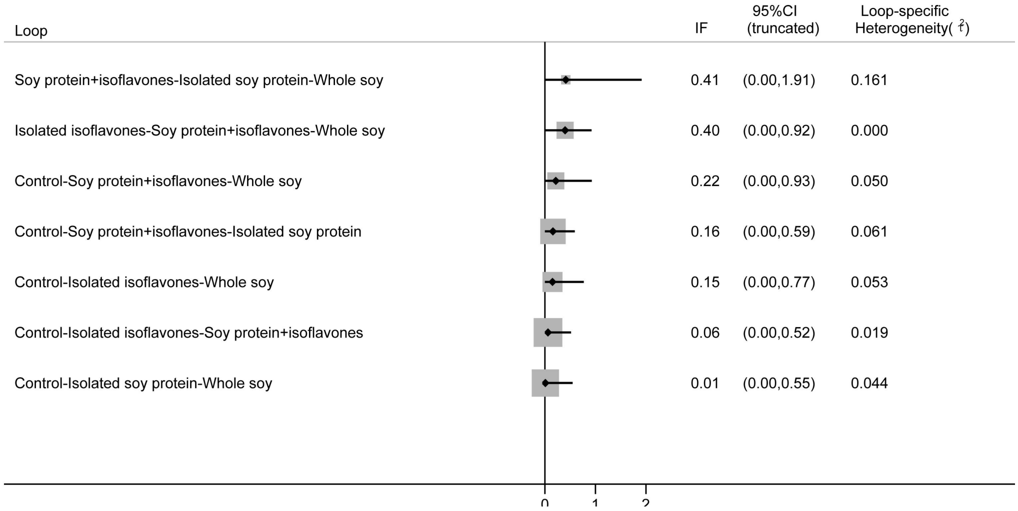

SUPPLEMENTAL FIGURE 8 Evaluation of inconsistency by using loop-specific approach for insulin. IFs are calculated as the absolute difference between direct and indirect estimates and therefore confidence intervals are truncated to 0. Loops that their lower CI limit does not reach the 0 line are considered to present statistically significant inconsistency. IF, inconsistency factor.

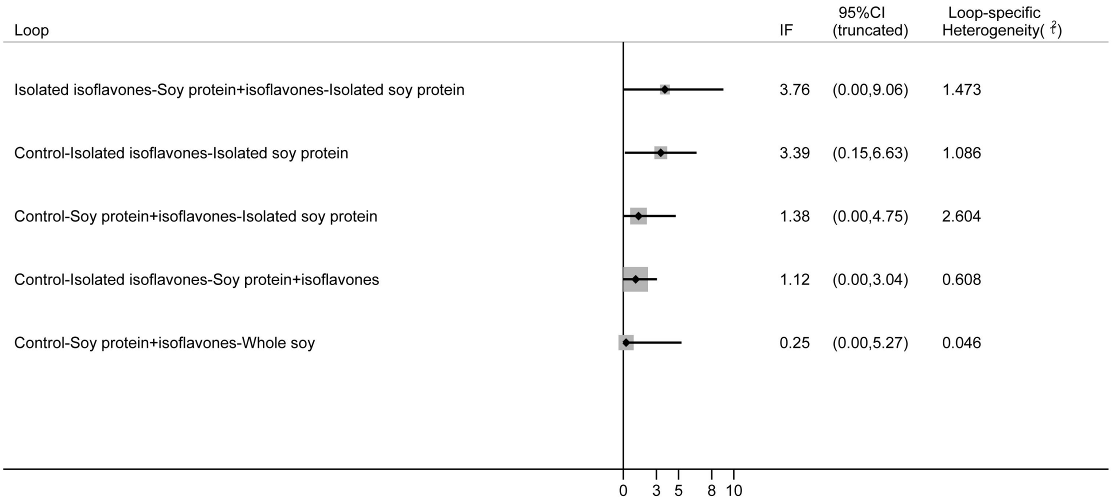

SUPPLEMENTAL FIGURE 9 Evaluation of inconsistency by using loop-specific approach for HOMA-IR. IFs are calculated as the absolute difference between direct and indirect estimates and therefore confidence intervals are truncated to 0. Loops that their lower CI limit does not reach the 0 line are considered to present statistically significant inconsistency. IF, inconsistency factor.

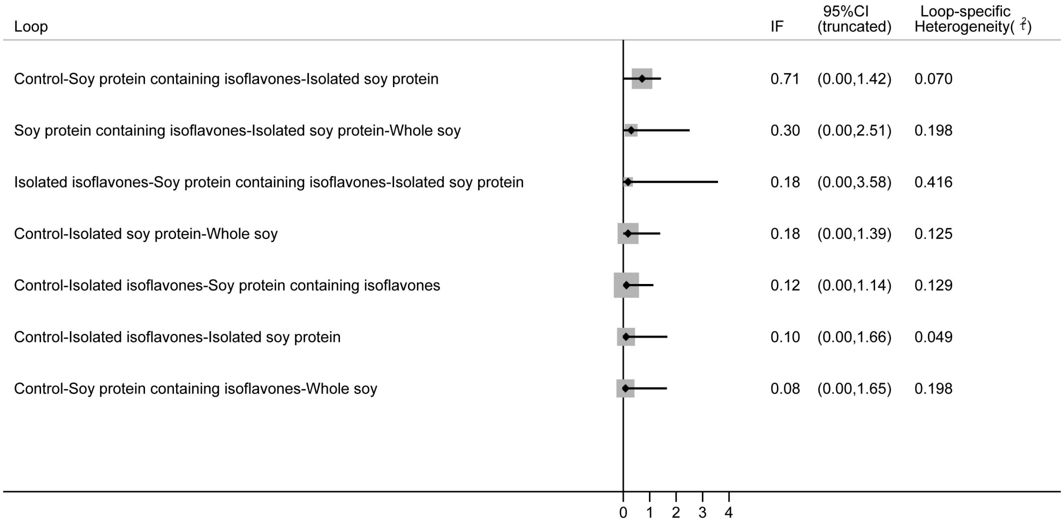

SUPPLEMENTAL FIGURE 10 Evaluation of inconsistency by using loop-specific approach for HbA1c. IFs are calculated as the absolute difference between direct and indirect estimates and therefore confidence intervals are truncated to 0. Loops that their lower CI limit does not reach the 0 line are considered to present statistically significant inconsistency. IF, inconsistency factor.

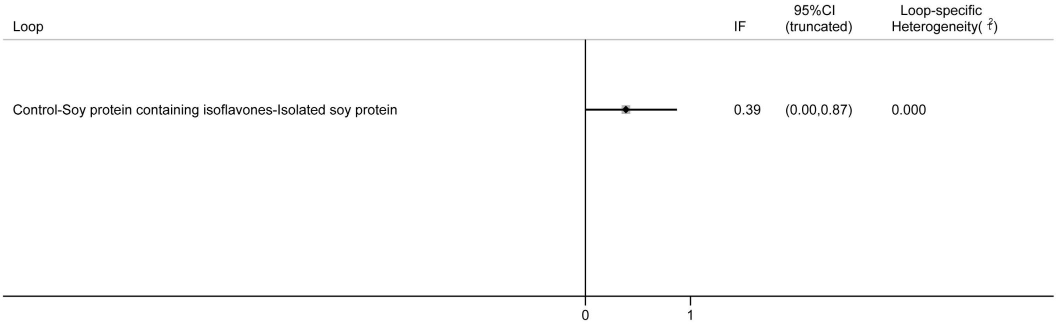

Supplemental Table 18 Overall GRADE quality of evidence for blood glucose from network meta-analysis.[1]

Comparisons	Study limitations	Imprecision	Inconsistency	Indirectness	Publication bias	Confidence in MD for overall change in blood glucose
Whole soy vs Control	15% of the estimate from studies at high risk, and 85% at moderate risk	MD -0.20, 95% CI (-0.33,-0.08)	No inconsistency between the direct and indirect estimate (Node-split *P*=0.74 and tau=0.11)	The treatment effects were not significantly influenced by clinical modifiers in the subgroup analyses	Undetectable by the routine method The comparison-adjusted funnel plot for the network is not suggestive of any dominant publication bias	Low
Soy protein vs Control	3% of the estimate from studies at high risk, and 97% at moderate risk	MD 0.10, 95% CI (-0.07,0.27)	No inconsistency between the direct and indirect estimate (Node-split *P*=0.47 and tau=0.10)	The treatment effects were not significantly influenced by clinical modifiers in the subgroup analyses	Undetectable by the routine method The comparison-adjusted funnel plot for the network is not suggestive of any dominant publication bias	Low
Soy protein+isoflavones vs Control	5% of the estimate from studies at high risk, and 95% at moderate risk	MD -0.04, 95% CI (-0.19,0.12)	No inconsistency between the direct and indirect estimate (Node-split *P*=0.98 and tau=0.11)	The treatment effects were not significantly influenced by clinical modifiers in the subgroup analyses	Undetectable by the routine method The comparison-adjusted funnel plot for the network is not suggestive of any dominant publication bias	Low
Isolated isoflavones vs Control	0% of the estimate from studies at high risk, and 100% at moderate risk	MD -0.16, 95% CI (-0.30,-0.02)	No inconsistency between the direct and indirect estimate (Node-split *P*=0.67 and tau=0.11)	The treatment effects were significantly influenced by some clinical modifiers in the subgroup analyses	Undetectable by the routine method The comparison-adjusted funnel plot for the network is not suggestive of any dominant publication bias	Moderate

Supplemental Table 18 Continued

Comparisons	Study limitations	Imprecision	Inconsistency	Indirectness	Publication bias	Confidence in MD for overall change in blood glucose
Whole soy vs Isolated soy protein	0% of the estimate from studies at high risk, and 100% at moderate risk	MD 0.30, 95% CI (0.10,0.50)	No inconsistency between the direct and indirect estimate (Node-split *P*=0.51 and tau=0.11)	The treatment effects were significantly influenced by some clinical modifiers in the subgroup analyses	Undetectable by the routine method The comparison-adjusted funnel plot for the network is not suggestive of any dominant publication bias	Moderate
Whole soy vs Soy protein+isoflavones	1% of the estimate from studies at high risk, and 99% at moderate risk	MD 0.17, 95% CI (-0.02,0.36)	-	The treatment effects were not significantly influenced by clinical modifiers in the subgroup analyses	Undetectable by the routine method The comparison-adjusted funnel plot for the network is not suggestive of any dominant publication bias	Low
Whole soy vs Isolated isoflavones	0% of the estimate from studies at high risk, and 100% at moderate risk	MD 0.05, 95% CI (-0.14,0.23)	No inconsistency between the direct and indirect estimate (Node-split *P*=0.60 and tau=0.10)	The treatment effects were not significantly influenced by clinical modifiers in the subgroup analyses	Undetectable by the routine method The comparison-adjusted funnel plot for the network is not suggestive of any dominant publication bias	Moderate
Isolated soy protein vs Soy protein+isoflavones	0% of the estimate from studies at high risk, and 100% at moderate risk	MD -0.13, 95% CI (-0.29,0.02)	No inconsistency between the direct and indirect estimate (Node-split *P*=0.98 and tau=0.11)	The treatment effects were not significantly influenced by clinical modifiers in the subgroup analyses	Undetectable by the routine method The comparison-adjusted funnel plot for the network is not suggestive of any dominant publication bias	Moderate

Supplemental Table 18 Continued

Comparisons	Study limitations	Imprecision	Inconsistency	Indirectness	Publication bias	Confidence in MD for overall change in blood glucose
Soy protein+isoflavones vs Isolated isoflavones	0% of the estimate from studies at high risk, and 100% at moderate risk	MD -0.12, 95% CI (-0.33,0.08)	-	The treatment effects were not significantly influenced by clinical modifiers in the subgroup analyses	Undetectable by the routine method The comparison-adjusted funnel plot for the network is not suggestive of any dominant publication bias	Very Low

[a] The quality of evidence of network estimates for all outcomes by using the GRADE framework, which characterizes the quality of a body of evidence on the basis of the study limitations, imprecision, inconsistency, indirectness, and publication bias

Supplemental Table 19 Overall GRADE quality of evidence for fasting insulin from network meta-analysis.[1]

Comparisons	Study limitations	Imprecision	Inconsistency	Indirectness	Publication bias	Confidence in MD for overall change in fasting insulin
Whole soy vs Control	10% of the estimate from studies at high risk, and 90% at moderate risk	MD -1.25, 95% CI (-2.35,-0.16)	No inconsistency between the direct and indirect estimate (Node-split *P*=0.33 and tau=1.59)	The treatment effects were significantly influenced by some clinical modifiers in the subgroup analyses	Undetectable by the routine method The comparison-adjusted funnel plot for the network is not suggestive of any dominant publication bias	Low
Soy protein vs Control	3% of the estimate from studies at high risk, and 97% at moderate risk	MD 0.46, 95% CI (-0.86,1.79)	No inconsistency between the direct and indirect estimate (Node-split *P*=0.35 and tau=1.58)	The treatment effects were not significantly influenced by clinical modifiers in the subgroup analyses	Undetectable by the routine method The comparison-adjusted funnel plot for the network is not suggestive of any dominant publication bias	Low
Soy protein+isoflavones vs Control	5% of the estimate from studies at high risk, and 95% at moderate risk	MD -0.52, 95% CI (-1.64,0.61)	No inconsistency between the direct and indirect estimate (Node-split *P*=0.45 and tau=1.59)	The treatment effects were not significantly influenced by clinical modifiers in the subgroup analyses	Undetectable by the routine method The comparison-adjusted funnel plot for the network is not suggestive of any dominant publication bias	Low

Supplemental Table 19 Continued

Comparisons	Study limitations	Imprecision	Inconsistency	Indirectness	Publication bias	Confidence in MD for overall change in fasting insulin
Isolated isoflavones vs Control	0% of the estimate from studies at high risk, and 100% at moderate risk	MD -0.92, 95% CI (-1.84,-0.01)	No inconsistency between the direct and indirect estimate (Node-split *P*=0.01 and tau=1.41)	The treatment effects were significantly influenced by some clinical modifiers in the subgroup analyses	Undetectable by the routine method The comparison-adjusted funnel plot for the network is not suggestive of any dominant publication bias	Very Low
Whole soy vs Soy protein+isoflavones	1% of the estimate from studies at high risk, and 99% at moderate risk	MD 0.74, 95% CI (-0.78,2.26)	No inconsistency between the direct and indirect estimate (Node-split *P*=0.70 and tau=1.61)	The treatment effects were not significantly influenced by clinical modifiers in the subgroup analyses	Undetectable by the routine method The comparison-adjusted funnel plot for the network is not suggestive of any dominant publication bias	Low
Isolated soy protein vs Soy protein+isoflavones	0% of the estimate from studies at high risk, and 100% at moderate risk	MD -0.98, 95% CI (-2.05,0.10)	No inconsistency between the direct and indirect estimate (Node-split *P*=0.61 and tau=1.61)	The treatment effects were not significantly influenced by clinical modifiers in the subgroup analyses	Undetectable by the routine method The comparison-adjusted funnel plot for the network is not suggestive of any dominant publication bias	Moderate

Supplemental Table 19 Continued

Comparisons	Study limitations	Imprecision	Inconsistency	Indirectness	Publication bias	Confidence in MD for overall change in fasting insulin
Isolated soy protein vs Isolated isoflavones	0% of the estimate from studies at high risk, and 100% at moderate risk	MD -1.38, 95% CI (-2.91,0.14)	No inconsistency between the direct and indirect estimate (Node-split *P*=0.00 and tau=1.34)	The treatment effects were not significantly influenced by clinical modifiers in the subgroup analyses	-Undetectable by the routine method The comparison-adjusted funnel plot for the network is not suggestive of any dominant publication bias	Very Low
Soy protein+isoflavones vs Isolated isoflavones	0% of the estimate from studies at high risk, and 100% at moderate risk	MD -0.41, 95% CI (-1.76,0.95)	No inconsistency between the direct and indirect estimate (Node-split *P*=0.57 and tau=1.61)	The treatment effects were not significantly influenced by clinical modifiers in the subgroup analyses	Undetectable by the routine method The comparison-adjusted funnel plot for the network is not suggestive of any dominant publication bias	Moderate

[a] The quality of evidence of network estimates for all outcomes by using the GRADE framework, which characterizes the quality of a body of evidence on the basis of the study limitations, imprecision, inconsistency, indirectness, and publication bias

Supplemental Table20 Overall GRADE quality of evidence for HOMA-IR from network meta-analysis.[1]

Comparisons	Study limitations	Imprecision	Inconsistency	Indirectness	Publication bias	Confidence in MD for overall change in HOMA-IR
Whole soy vs Control	8% of the estimate from studies at high risk, and 92% at moderate risk	MD -0.51, 95% CI (-0.82,-0.19)	No inconsistency between the direct and indirect estimate (Node-split *P*=0.36 and tau=0.44)	The treatment effects were significantly influenced by some clinical modifiers in the subgroup analyses	Undetectable by the routine method The comparison-adjusted funnel plot for the network is not suggestive of any dominant publication bias	Low
Soy protein vs Control	1% of the estimate from studies at high risk, and 99% at moderate risk	MD -0.16, 95% CI (-0.55,0.23)	No inconsistency between the direct and indirect estimate (Node-split *P*=0.72 and tau=0.43)	The treatment effects were not significantly influenced by clinical modifiers in the subgroup analyses	Undetectable by the routine method The comparison-adjusted funnel plot for the network is not suggestive of any dominant publication bias	Low
Soy protein+isoflavones vs Control	1% of the estimate from studies at high risk, and 99% at moderate risk	MD -0.50, 95% CI (-0.85,-0.14)	No inconsistency between the direct and indirect estimate (Node-split *P*=0.64 and tau=0.43)	The treatment effects were significantly influenced by some clinical modifiers in the subgroup analyses	Undetectable by the routine method The comparison-adjusted funnel plot for the network is not suggestive of any dominant publication bias	Low

Supplemental Table 20 Continued

Comparisons	Study limitations	Imprecision	Inconsistency	Indirectness	Publication bias	Confidence in MD for overall change in HOMA-IR
Isolated isoflavones vs Control	0% of the estimate from studies at high risk, and 100% at moderate risk	MD -0.22, 95% CI (-0.54,-0.10)	No inconsistency between the direct and indirect estimate (Node-split *P*=0.31 and tau=0.43)	The treatment effects were not significantly influenced by clinical modifiers in the subgroup analyses	Undetectable by the routine method The comparison-adjusted funnel plot for the network is not suggestive of any dominant publication bias	High
Whole soy vs Soy protein+isoflavones	1% of the estimate from studies at high risk, and 99% at moderate risk	MD 0.01, 95% CI (-0.44,0.46)	No inconsistency between the direct and indirect estimate (Node-split *P*=0.73 and tau=0.45)	The treatment effects were not significantly influenced by clinical modifiers in the subgroup analyses	Undetectable by the routine method The comparison-adjusted funnel plot for the network is not suggestive of any dominant publication bias	Low
Isolated soy protein vs Soy protein+isoflavones	0% of the estimate from studies at high risk, and 100% at moderate risk	MD -0.34, 95% CI (-0.73,0.06)	No inconsistency between the direct and indirect estimate (Node-split *P*=0.60 and tau=0.43)	The treatment effects were not significantly influenced by clinical modifiers in the subgroup analyses	Undetectable by the routine method The comparison-adjusted funnel plot for the network is not suggestive of any dominant publication bias	Moderate

Supplemental Table 20 Continued

Comparisons	Study limitations	Imprecision	Inconsistency	Indirectness	Publication bias	Confidence in MD for overall change in HOMA-IR
Isolated soy protein vs Isolated isoflavones	0% of the estimate from studies at high risk, and 100% at moderate risk	MD -0.06, 95% CI (-0.55,0.43)	-	The treatment effects were not significantly influenced by clinical modifiers in the subgroup analyses	-Undetectable by the routine method The comparison-adjusted funnel plot for the network is not suggestive of any dominant publication bias	Very Low
Soy protein+isoflavones vs Isolated isoflavones	0% of the estimate from studies at high risk, and 100% at moderate risk	MD 0.28, 95% CI (-0.18,0.73)	No inconsistency between the direct and indirect estimate (Node-split *P*=0.66 and tau=0.45)	The treatment effects were not significantly influenced by clinical modifiers in the subgroup analyses	Undetectable by the routine method The comparison-adjusted funnel plot for the network is not suggestive of any dominant publication bias	Moderate

[a] The quality of evidence of network estimates for all outcomes by using the GRADE framework, which characterizes the quality of a body of evidence on the basis of the study limitations, imprecision, inconsistency, indirectness, and publication bias.

Supplemental Table 21 Overall GRADE quality of evidence for HbA1c from network meta-analysis.[1]

Comparisons	Study limitations	Imprecision	Inconsistency	Indirectness	Publication bias	Confidence in MD for overall change in HbA1c
Whole soy vs Control	0% of the estimate from studies at high risk, and 100% at moderate risk	MD 0.00, 95% CI (-0.71,0.71)	-	-	Undetectable by the routine method The comparison-adjusted funnel plot for the network is not suggestive of any dominant publication bias	Low
Soy protein vs Control	0% of the estimate from studies at high risk, and 100% at moderate risk	MD -0.04, 95% CI (-0.28,0.19)	No inconsistency between the direct and indirect estimate (Node-split *P*=0.12 and tau=4.01e-07)	-	Undetectable by the routine method The comparison-adjusted funnel plot for the network is not suggestive of any dominant publication bias	Very Low
Soy protein+isoflavones vs Control	1% of the estimate from studies at high risk, and 99% at moderate risk	MD -0.02, 95% CI (-0.21,0.17)	No inconsistency between the direct and indirect estimate (Node-split *P*=0.12 and tau=6.24e-08)	-	Undetectable by the routine method The comparison-adjusted funnel plot for the network is not suggestive of any dominant publication bias	Very Low

Supplemental Table 21 Continued

Comparisons	Study limitations	Imprecision	Inconsistency	Indirectness	Publication bias	Confidence in MD for overall change in HbA1c
Isolated isoflavones vs Control	0% of the estimate from studies at high risk, and 100% at moderate risk	MD -0.23, 95% CI (-0.23,0.23)	-	-	Undetectable by the routine method The comparison-adjusted funnel plot for the network is not suggestive of any dominant publication bias	Very Low
Isolated soy protein vs Soy protein+isoflavones	0% of the estimate from studies at high risk, and 100% at moderate risk	MD 0.03, 95% CI (-0.16,0.21)	No inconsistency between the direct and indirect estimate (Node-split P=0.12 and tau=1.02e-06)	-	Undetectable by the routine method The comparison-adjusted funnel plot for the network is not suggestive of any dominant publication bias	Very Low

[a] The quality of evidence of network estimates for all outcomes by using the GRADE framework, which characterizes the quality of a body of evidence on the basis of the study limitations, imprecision, inconsistency, indirectness, and publication bias.

SUPPLEMENTAL TABLE 22 Results of sensitivity analysis after excluding the studies with crossover designs for blood glucose.[1]

	Control	Whole soy	Soy protein	Soy protein + isoflavones	Isoflavones
Control	-	**-0.21 (-0.37,-0.04)**	0.09 (-0.12,0.30)	-0.03 (-0.21,0.15)	**-0.18 (-0.35,-0.02)**
Whole soy		-	**0.29 (0.04,0.55)**	0.18 (-0.06,0.41)	0.02 (-0.20,0.25)
Soy protein			-	-0.12 (-0.31,0.07)	**-0.27 (-0.53,-0.01)**
Soy protein + isoflavones				-	-0.15 (-0.39,0.09)
Isoflavones					-

[1]The values below the diets correspond to the mean differences with 95% confidence intervals between the column and the row

SUPPLEMENTAL TABLE 23 Results of sensitivity analysis after excluding the studies with crossover designs for insulin.[1]

	Control	Whole soy	Soy protein	Soy protein + isoflavones	Isoflavones
Control	-	**-1.45 (-2.68,-0.22)**	0.65 (-0.82,2.13)	-0.62 (-1.87,0.62)	**-1.10 (-2.10,-0.09)**
Whole soy		-	**2.11 (0.22,3.99)**	0.83 (-0.86,2.52)	0.35 (-1.22,1.93)
Soy protein			-	-1.28 (-2.55,0.00)	-1.75 (-3.43,-0.07)
Soy protein + isoflavones				-	-0.47 (-1.96,1.01)
Isoflavones					-

[1]The values below the diets correspond to the mean differences with 95% confidence intervals between the column and the row

SUPPLEMENTAL TABLE 24 Results of sensitivity analysis after excluding the studies with crossover designs for HOMA-IR.[1]

	Control	Whole soy	Soy protein	Soy protein + isoflavones	Isoflavones
Control	**-**	**-0.51 (-0.88,-0.15)**	-0.17 (-0.64,0.31)	**-0.45 (-0.84,-0.05)**	-0.25 (-0.62,0.11)
Whole soy		-	0.35 (-0.24,0.94)	0.06 (-0.45,0.58)	0.26 (-0.25,0.78)
Soy protein			-	-0.28 (-0.74,0.17)	-0.09 (-0.67,0.50)
Soy protein + isoflavones				-	0.20 (-0.31,0.71)
Isoflavones					-

[1]The values below the diets correspond to the mean differences with 95% confidence intervals between the column and the row

SUPPLEMENTAL TABLE 25. Results of sensitivity analysis performed based on the studies with a normal blood glucose mean baseline for fasting glucose.[1]

	Control	Whole soy	Soy protein	Soy protein + isoflavones	Isoflavones
Control	-	**-0.11 (-0.22,-0.00)**	0.05 (-0.11,0.20)	-0.02 (-0.15,0.12)	**-0.16 (-0.28,-0.05)**
Whole soy		-	0.16 (-0.03,0.34)	0.09 (-0.07,0.26)	-0.05 (-0.20,0.10)
Soy protein			-	-0.06 (-0.19,0.07)	**-0.21 (-0.40,-0.02)**
Soy protein + isoflavones				-	-0.15 (-0.32,0.03)
Isoflavones					-

[1]Results of sensitivity analysis performed based on the studies with a normal blood glucose mean baseline for fasting glucose

SUPPLEMENTAL TABLE 26 Results of sensitivity analysis performed based on the studies with a normal blood glucose mean baseline for insulin.[1]

	Control	Whole soy	Soy protein	Soy protein + isoflavones	Isoflavones
Control	-	-0.09 (-1.30,1.12)	0.77 (-0.58,2.12)	0.03 (-1.20,1.26)	-1.02 (-1.90,-0.14)
Whole soy		-	0.86 (-0.95,2.68)	0.12 (-1.61,1.85)	-0.93 (-2.43,0.56)
Soy protein			-	-0.74 (-1.73,0.24)	-1.79 (-3.32,-0.27)
Soy protein + isoflavones				-	-1.05 (-2.48,0.38)
Isoflavones					-

[1]The values below the diets correspond to the mean differences with 95% confidence intervals between the column and the row

SUPPLEMENTAL TABLE 27 Results of sensitivity analysis performed based on the studies with a normal blood glucose mean baseline for HOMA-IR.[1]

	Control	Whole soy	Soy protein	Soy protein + isoflavones	Isoflavones
Control	-	-0.11 (-0.45,0.22)	-0.12 (-0.55,0.32)	**-0.48 (-0.85,-0.11)**	-0.23 (-0.52,0.06)
Whole soy		-	-0.00 (-0.55,0.54)	-0.37 (-0.87,0.13)	-0.12 (-0.56,0.33)
Soy protein			-	-0.36 (-0.74,0.01)	-0.11 (-0.63,0.40)
Soy protein + isoflavones				-	0.25 (-0.22,0.72)
Isoflavones					-

[1]The values below the diets correspond to the mean differences with 95% confidence intervals between the column and the row

SUPPLEMENTAL TABLE 28 Results of sensitivity analysis performed based on the studies with a abnormal blood glucose mean baseline for fasting glucose.[1]

	Control	Whole soy	Soy protein	Soy protein + isoflavones	Isoflavones
Control	**-**	**-0.68 (-1.34,-0.03)**	0.23 (-0.61,1.07)	-0.22 (-0.96,0.52)	-0.26 (-1.01,0.50)
Whole soy		-	0.92 (-0.06,1.89)	0.46 (-0.50,1.42)	0.43 (-0.56,1.41)
Soy protein			-	-0.45 (-1.35,0.44)	-0.49 (-1.58,0.60)
Soy protein + isoflavones				-	-0.04 (-1.01,0.93)
Isoflavones					-

[1]The values below the diets correspond to the mean differences with 95% confidence intervals between the column and the row

SUPPLEMENTAL TABLE 29 Results of sensitivity analysis performed based on the studies with a abnormal blood glucose mean baseline for insulin.[1]

	Control	Whole soy	Soy protein	Soy protein + isoflavones	Isoflavones
Control	-	**-3.43 (-5.04,-1.81)**	0.84 (-2.92,4.61)	-1.98 (-3.81,-0.15)	-0.59 (-2.71,1.53)
Whole soy		-	**4.27 (0.21,8.33)**	1.44 (-0.79,3.68)	**2.83 (0.21,5.45)**
Soy protein			-	-2.82 (-6.86,1.21)	-1.44 (-5.73,2.86)
Soy protein + isoflavones				-	1.39 (-1.08,3.85)
Isoflavones					-

[1]The values below the diets correspond to the mean differences with 95% confidence intervals between the column and the row

SUPPLEMENTAL TABLE 30 Results of sensitivity analysis performed based on the studies with a abnormal blood glucose mean baseline for HOMA-IR.[1]

	Control	Whole soy	Soy protein	Soy protein + isoflavones	Isoflavones
Control	-	**-1.12 (-1.65,-0.58)**	-0.53 (-1.23,0.18)	-0.55 (-1.27,0.17)	-0.21 (-1.00,0.59)
Whole soy		-	0.59 (-0.18,1.35)	0.57 (-0.23,1.37)	0.91 (-0.02,1.84)
Soy protein			-	-0.02 (-1.00,0.96)	0.32 (-0.74,1.37)
Soy protein + isoflavones				-	0.34 (-0.59,1.28)
Isoflavones					-

[1]The values below the diets correspond to the mean differences with 95% confidence intervals between the column and the row

SUPPLEMENTAL TABLE 31 Results of sensitivity analysis based on the studies with a dyslipidemic mean baseline for blood glucose.[1]

	Control	Whole soy	Soy protein	Soy protein + isoflavones	Isoflavones
Control	**-**	**-0.18 (-0.29,-0.07)**	0.05 (-0.10,0.21)	0.01 (-0.14,0.16)	-0.10 (-0.23,0.03)
Whole soy		-	0.23 (0.06,0.40)	0.19 (0.02,0.36)	0.08 (-0.09,0.24)
Soy protein			-	-0.04 (-0.20,0.11)	-0.16 (-0.36,0.04)
Soy protein + isoflavones				-	-0.11 (-0.31,0.08)
Isoflavones					-

[1]The values below the diets correspond to the mean differences with 95% confidence intervals between the column and the row

SUPPLEMENTAL TABLE 32 Results of sensitivity analysis based on the studies with a dyslipidemic mean baseline for insulin.[1]

	Control	Whole soy	Soy protein	Soy protein + isoflavones	Isoflavones
Control	-	-0.86 (-1.92,0.21)	-0.14 (-1.62,1.35)	-1.22 (-2.47,0.03)	-0.45 (-1.41,0.51)
Whole soy		-	0.72 (-1.08,2.52)	-0.37 (-1.95,1.22)	0.41 (-1.03,1.85)
Soy protein			-	-1.09 (-2.36,0.19)	-0.31 (-2.08,1.46)
Soy protein + isoflavones				-	0.77 (-0.80,2.35)
Isoflavones					-

[1]The values below the diets correspond to the mean differences with 95% confidence intervals between the column and the row

SUPPLEMENTAL TABLE 33 Results of sensitivity analysis based on the studies with a dyslipidemic mean baseline for HOMA-IR.[1]

	Control	Whole soy	Soy protein	Soy protein + isoflavones	Isoflavones
Control	**-**	**-0.27 (-0.52,-0.02)**	-0.13 (-0.45,0.19)	**-0.68 (-1.02,-0.35)**	-0.03 (-0.36,0.29)
Whole soy		-	0.14 (-0.23,0.51)	-0.42 (-0.81,-0.03)	0.24 (-0.17,0.64)
Soy protein			-	-0.55 (-0.92,-0.19)	0.10 (-0.35,0.55)
Soy protein + isoflavones				-	0.65 (0.19,1.11)
Isoflavones					-

[1]The values below the diets correspond to the mean differences with 95% confidence intervals between the column and the row

SUPPLEMENTAL TABLE 34 Results of sensitivity analysis performed based on the studies with overweight or obesity participants for blood glucose.[1]

	Control	Whole soy	Soy protein	Soy protein + isoflavones	Isoflavones
Control	**-**	**-0.18 (-0.34,-0.02)**	0.10 (-0.10,0.29)	-0.04 (-0.21,0.14)	-0.12 (-0.29,0.05)
Whole soy		-	**0.28 (0.04,0.52)**	0.14 (-0.08,0.37)	0.06 (-0.17,0.29)
Soy protein			-	-0.14 (-0.30,0.03)	-0.22 (-0.47,0.04)
Soy protein + isoflavones				-	-0.08 (-0.32,0.16)
Isoflavones					-

[1]The values below the diets correspond to the mean differences with 95% confidence intervals between the column and the row

SUPPLEMENTAL TABLE 35 Results of sensitivity analysis performed based on the studies with overweight or obesity participants for insulin.[1]

	Control	Whole soy	Soy protein	Soy protein + isoflavones	Isoflavones
Control	-	-0.53 (-1.93,0.88)	0.47 (-0.84,1.79)	-0.48 (-1.63,0.68)	**-1.05 (-2.04,-0.06)**
Whole soy		-	1.00 (-0.89,2.88)	0.05 (-1.70,1.79)	-0.52 (-2.24,1.19)
Soy protein			-	-0.95 (-1.99,0.10)	-1.52 (-3.09,0.05)
Soy protein + isoflavones				-	-0.57 (-2.02,0.88)
Isoflavones					-

[1]The values below the diets correspond to the mean differences with 95% confidence intervals between the column and the row

SUPPLEMENTAL TABLE 36 Results of sensitivity analysis performed based on the studies with overweight or obesity participants for HOMA-IR.[1]

	Control	Whole soy	Soy protein	Soy protein + isoflavones	Isoflavones
Control	-	-0.27 (-0.62,0.07)	-0.16 (-0.56,0.23)	**-0.51 (-0.84,-0.18)**	-0.20 (-0.52,0.12)
Whole soy		-	0.11 (-0.39,0.61)	-0.24 (-0.69,0.21)	0.08 (-0.39,0.54)
Soy protein			-	-0.35 (-0.71,0.01)	-0.03 (-0.53,0.47)
Soy protein + isoflavones				-	0.31 (-0.14,0.76)
Isoflavones					-

[1]The values below the diets correspond to the mean differences with 95% confidence intervals between the column and the row

SUPPLEMENTAL FIGURE 11 Comparison-adjusted funnel plot for fasting glucose.[1]

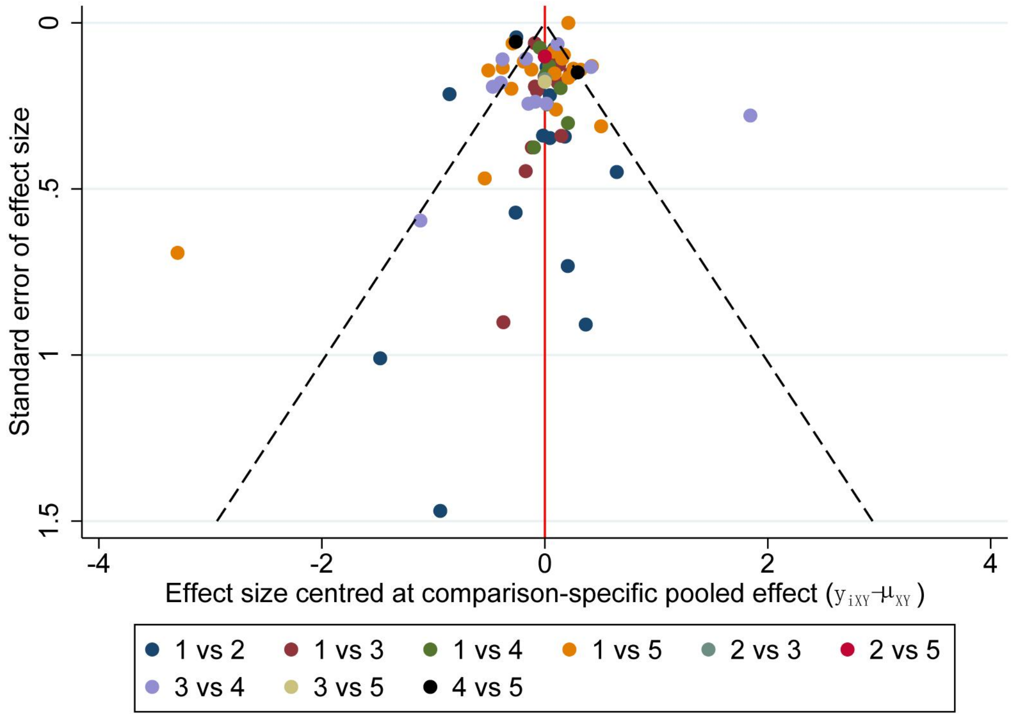

[1] 1=Control, 2= Isolated isoflavones, 3= Soy protein + isoflavones, 4= Soy protein, 5= Whole soy

SUPPLEMENTAL FIGURE 12 Comparison-adjusted funnel plot for fasting insulin.[1]

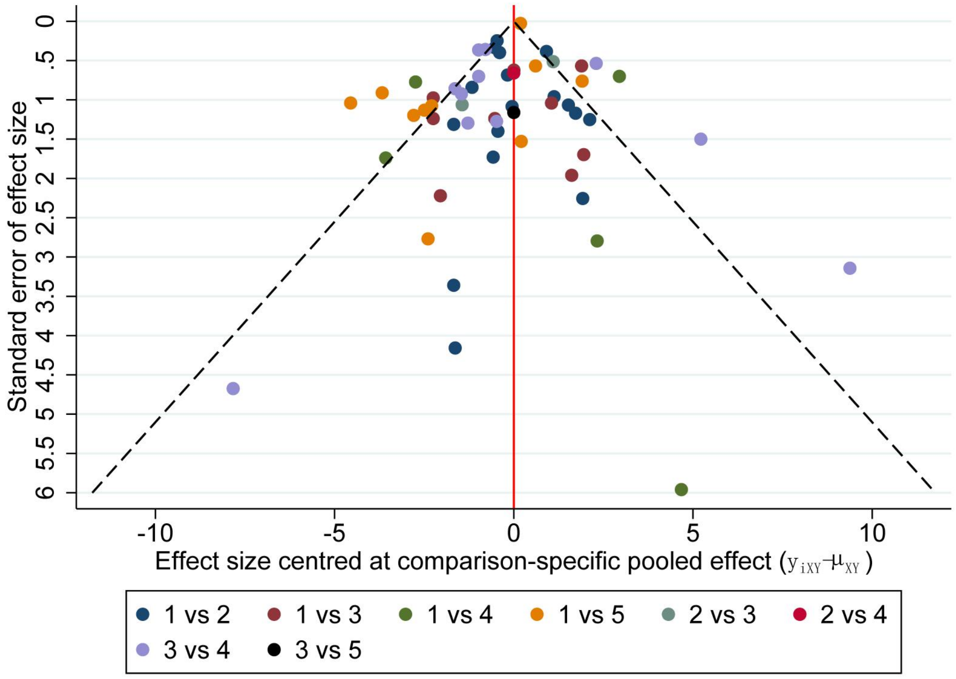

[1]1=Control, 2= Isolated isoflavones, 3= Soy protein + isoflavones, 4= Soy protein, 5= Whole soy

SUPPLEMENTAL FIGURE 13 Comparison-adjusted funnel plot for HOMA-IR.[1]

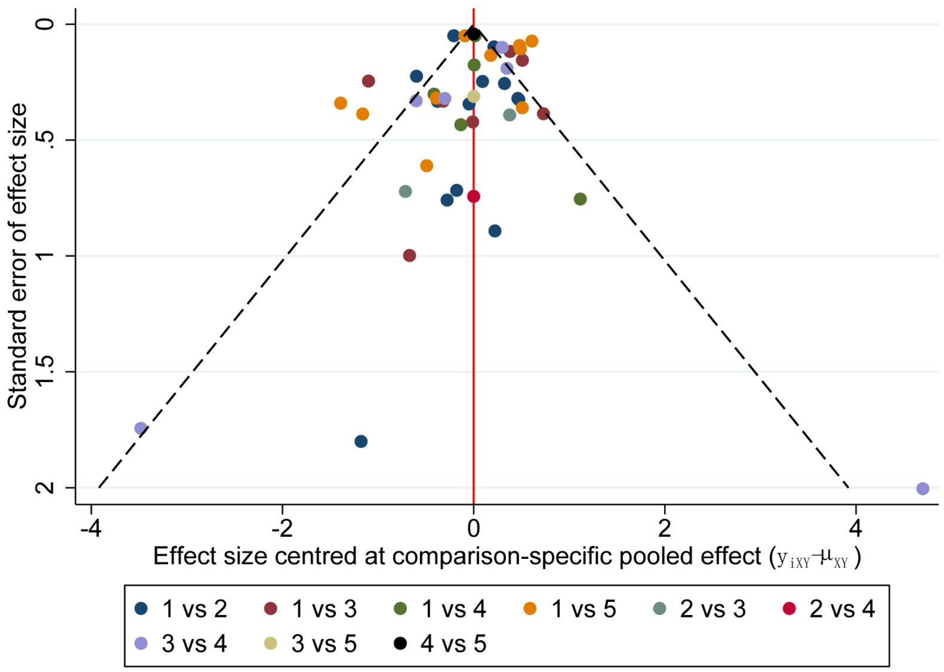

[1] 1=Control, 2= Isolated isoflavones, 3= Soy protein + isoflavones, 4= Soy protein, 5= Whole soy

SUPPLEMENTAL FIGURE 14 Comparison-adjusted funnel plot for HbA1C.[1]

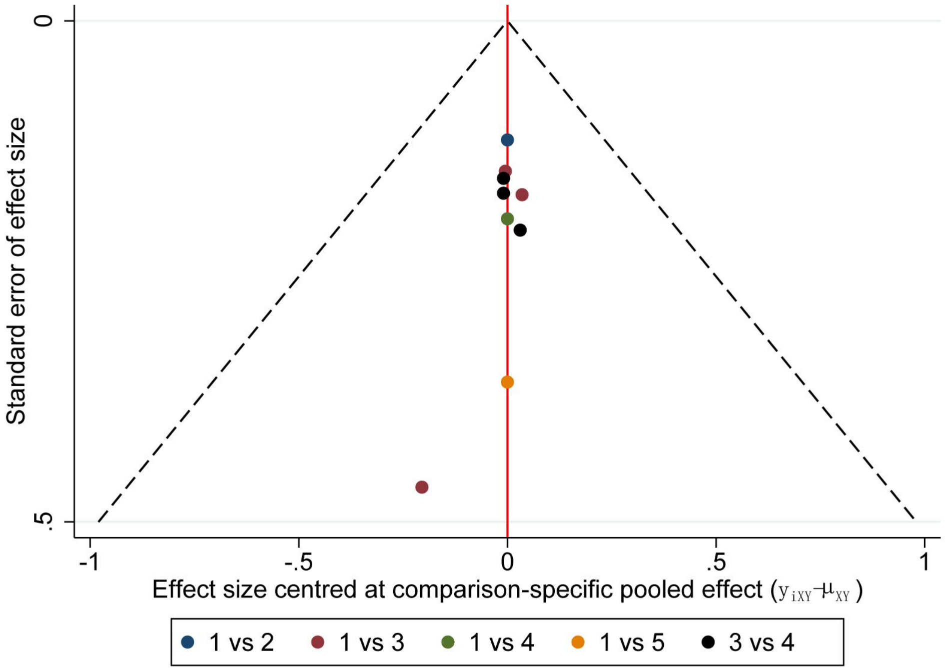

[1]1=Control, 2= Isolated isoflavones, 3= Soy protein + isoflavones, 4= Soy protein, 5= Whole soy
